# Interpretable multi-omics machine learning reveals drought-driven shifts in plant-microbe interactions

**DOI:** 10.1186/s40793-026-00883-x

**Published:** 2026-04-10

**Authors:** Hayato Yoshioka, Pavla Debeljak, Soizic Prado, Yushiro Fuji, Yasunori Ichihashi, Hiroyoshi Iwata

**Affiliations:** 1 Department of Agricultural and Environmental Biology, Graduate School of Agricultural and Life Science, The University of Tokyo, Tokyo, Japan; 2 Muséum National d’Histoire Naturelle, Unité Molécules de Communication et Adaptation des Micro-Organismes, UMR 7245, CNRS, Sorbonne Université, Paris, France; 3 SupBiotech, Villejuif, France; 4 Center for Sustainable Resource Science, RIKEN, Yokohama, Japan; 5 Center for Sustainable Resource Science, RIKEN, Tsukuba, Japan

## Abstract

****Background**:**

Plant-microbe interactions in the rhizosphere are central to plant growth, nutrient acquisition, and stress resilience. Although multi-omics approaches enable comprehensive profiling of different biological layers, integrating these data to understand the mechanisms underlying plant-microbe symbiosis, particularly under drought stress, remains a challenge.

****Results**:**

Genomic, metabolomic, and microbiome data from 198 soybean accessions grown under both control and drought conditions were integrated to identify environment-specific predictive features of the plant phenotypes. We compared best linear unbiased prediction (BLUP), genome-wide association study (GWAS), and a nonlinear machine learning model to evaluate their ability to detect informative features. The machine learning models provided flexible variable selection and outperformed linear models in capturing nonlinear dependencies. Model interpretation using SHapley Additive exPlanations (SHAP) indicated that the isoflavone derivative, daidzin, and the drought-tolerant *Candidatus Nitrosocosmicus*, were major contributors to phenotypic variation, specifically under drought stress. SHAP-based interaction networks indicated cross-omics links, including connections between daidzin, gamma-aminobutyric acid (GABA), and *Paenibacillus*.

****Conclusion**:**

The proposed interpretable machine learning approach for plant phenotype prediction identified multi-omics biomarkers and interactions, providing insights into plant adaptation to drought stress through environment-dependent rhizosphere networks and symbiotic associations.

**Supplementary Information:**

The online version contains supplementary material available at 10.1186/s40793-026-00883-x.

## Background

Interactions between plants and microbes are pivotal in regulating plant growth [39, 45]. In particular, rhizosphere microorganisms are crucial for plant health as they enhance nutrient uptake and strengthen plant resilience, especially under abiotic stresses, such as drought [2, 16]. Therefore, understanding the rhizosphere microbiome is essential for deciphering the mechanisms underlying plant-microorganism symbiosis, particularly those related to drought tolerance.

Recent advances in multi-omics technologies have enabled the concurrent profiling of genomic [15], metabolomic [10], and microbiome data [20]. These inter-omics datasets provide new opportunities to unravel the complex relationships among various biological systems. However, the integration and interpretation of such diverse data types pose significant computational and statistical challenges.

Best Linear Unbiased Prediction (BLUP) is a widely used linear mixed model in plant and animal breeding [21, 41]. It leverages genome-wide markers to predict phenotypic traits by capturing linear additive genetic contributions. While BLUP can incorporate multiple omics layers through multi-kernel approaches, it remains limited to modeling linear relationships among features.

Genome-wide association studies (GWAS) are popular for identifying individual SNP markers associated with phenotypic variation [3, 24]. However, GWAS typically analyzes genome-wide markers in isolation. When evaluating the significance of the interactions between genome-wide markers and other omics variables, the number of possible combinations and multiple testing burden may increase exponentially, making such analyses infeasible.

In contrast, machine learning approaches, including Random Forest (RF; [5]), are capable of nonlinear modeling and feature selection in multi-omics contexts. RF constructs a collection of decision trees and aggregates their predictions, providing both high predictive performance and feature importance scores for biological features [1, 27]. Nonlinear dependencies across omics layers have been observed in integrative studies [33, 50]. Recently, our group demonstrated that the RF model can effectively capture such complexity and outperform traditional linear models, such as BLUP, in predictive tasks when nonlinearity exists in multi-omics interactions [47].

SHapley Additive exPlanations (SHAP) is a game-theoretic approach that quantifies the marginal contribution of each feature by comparing model predictions across all possible subsets of input features [19]. Unlike traditional feature importance scores, SHAP values are both model-specific and globally consistent, offering insights into the interactions among features and their influence on predictions [18].

Recent studies have employed RF and SHAP to interpret phenotype prediction models, including those used for flowering time [43]. In addition, SHAP has been used with microbiome data to identify features distinguishing between drought and control conditions [11].

However, to date, no study has systematically integrated metabolomic, microbiomic, and genomic data to identify interacting biomarkers that bridge plant–microbe interactions. Moreover, the application of SHAP-based interpretability to uncover such cross-domain interactions in plant–microbe multi-omics datasets remains unexplored.

Due to the high-dimensional nature of the interaction analysis, we employed XGBoost [6], an ensemble tree-based gradient boosting method. Unlike RF, which aggregates independently grown trees, the gradient boosting model constructs trees sequentially by optimising a loss function, enabling efficient computation in high-dimensional settings.

In this study, we specifically focused on drought-induced shifts in plant-microbe interactions. By comparing plants grown under drought and control conditions, we aimed to capture how the plant-microbe-metabolome network collectively responds to water deficit stress.

Our analysis was conducted in three steps. First, we compared the BLUP and RF models to assess their respective abilities and identify biologically meaningful features from multi-omics datasets. Second, we employed SHAP to elucidate condition-specific features and cross-omics interactions that drive phenotypic variation. Finally, through this integrative multi-omics framework, we identify drought-responsive biomarkers and uncover condition-specific regulatory mechanisms that underpin adaptive responses in plant-soil microbiome systems.

## Methods

### Soybean multi-omics data

**Fig. 1:**
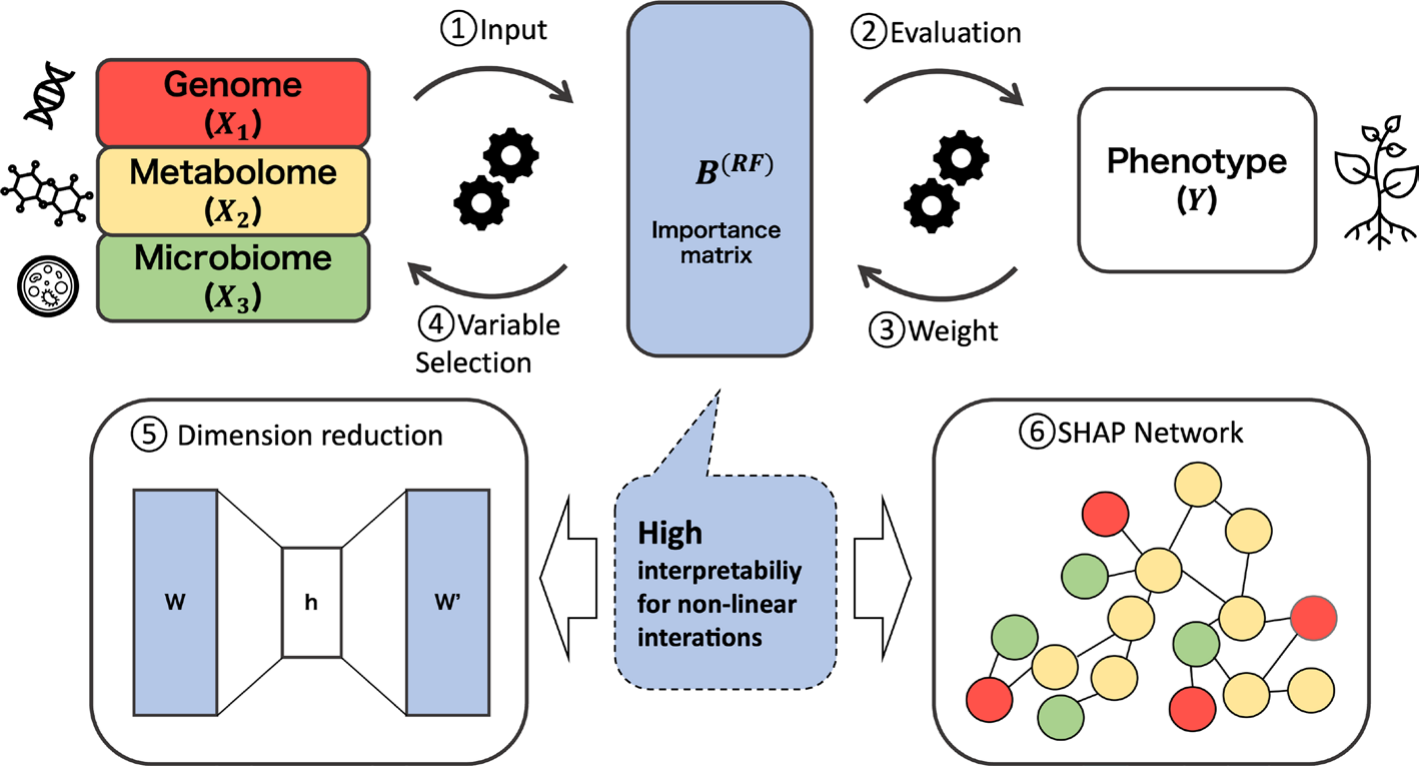
Concept of our framework. (1) Multi-omics data (Genome, Metabolome, Microbiome) are integrated as a single matrix and introduced into a soybean phenotype prediction model. (2) Phenotype (Y) is predicted using RF. (3) The model is analyzed retrospectively from the output Y to optimize the weights (importance: $B^{\text {(RF)}}$) of predictive factors within the omics data. (4) Variable selection is performed based on the nonlinear matrix $B^{\text {(RF)}}$, and a new prediction model is built using the selected variables. (5) Relationships among omics layers are interpreted in a low-dimensional latent space. (6) SHAP for network analysis of omics factors

We analysed multi-omics datasets collected from a common panel of $N = 198$ soybean accessions (Fig. 1). All omics and phenotype measurements were obtained from field trials conducted in 2019 under two watering conditions: watering (control) and non-watering (drought) to assess the effects of drought stress [31, 38]. The datasets included the following:

- *Whole-genome SNP genotypes (genome)* a $198 \times 425{,}858$ matrix, where each column corresponds to a single nucleotide polymorphism (SNP) marker in the soybean genome [15].
- *Rhizosphere metabolome* a $198 \times 263$ matrix of metabolome features obtained from rhizosphere samples. Each column represents the normalized peak area of a distinct metabolome feature detected by tandem mass spectrometry [7].
- *Rhizosphere microbiota profiles (microbiome)* a $198 \times 16{,}457$ matrix generated by 16 S rRNA gene amplicon sequencing of DNA extracted from rhizosphere samples. Each column represents the relative abundance of a bacterial taxon [7].
- *Plant phenotype* a $198 \times 9$ matrix. These traits include biomass-related features such as shoot and leaf fresh/dry weights, growth stage, plant height, main stem length, number of nodes, and number of branches.

The soybean accessions used in this study were selected from the Global Soybean Minicore Collection [15]. Furthermore, all rhizosphere microbiota profiles (microbiome) and rhizosphere metabolome data were collected from the same field plot as the phenotypic measurements. This design ensured consistency across samples and enabled integrative modeling across multiple omics layers.

To reduce the computational burden in the SHAP analysis of the RF model, SNPs were pruned based on linkage disequilibrium (LD). A representative subset of 3078 SNPs (LD threshold = 0.001) was selected from an initial set of 425,858 SNPs (LD threshold = 0.95). Despite the relatively small size compared with the full data, the 3,078-SNP subset provided approximate coverage of the entire genome loci (Fig. 5). Additionally, the 3,078-SNP subset achieved predictive performance comparable to that of larger sets containing approximately 10 to 34 k SNPs in phenotypic predictions (Fig. 6). The objective was to evaluate variables rather than optimise predictive performance. Thus, considering interpretability, computational limits in RF, and the need to balance the number of variables with those in the microbiome and metabolome datasets, we selected the 3,078-SNP set for subsequent RF analysis.

In the field trial, both microbiome and metabolome datasets were obtained from the same plots used for the phenotypic measurements. Note that these datasets were also recently utilized in Dang et al. [7]. Although they were collected from the same field, in the present study, the microbiome data were newly analyzed and integrated according to our experimental design, while the metabolome data were derived from those previously processed in Dang et al. [7].

The observed phenotypic values and omics features were filtered to remove missing values. After filtering, the dataset sizes (accessions $\times$ features) were as follows. For the control condition: metabolome (182 $\times$ 255), microbiome (182 $\times$ 5424), and phenotype (182 $\times$ 9). For the drought condition: metabolome (179 $\times$ 253), microbiome (179 $\times$ 4771), and phenotype (179 $\times$ 9). All datasets were subsequently scaled prior to modeling. Details of the omics data processing for integration are provided in Appendix A.

See the supplementary information in Appendix A for details on data collection and experimental conditions. The soybean raw genome sequences are available in the DDBJ Sequence Read Archive (DRA) under the BioProject accession number PRJDB7281, as previously processed and reported by Kajiya-Kanegae et al. [15]. The microbiome raw read sequences were originally processed and are available in the DDBJ DRA under the BioProject accession number PRJDB37881. The metabolome raw data were previously processed and analyzed by Dang et al. [7], and are available in the RIKEN DropMet database (http://prime.psc.riken.jp/menta.cgi/prime/drop_index; ID: DM0071). The plant phenotype data were originally processed and analyzed in this study. The microbiome, metabolome, and phenotype data are also provided as supplementary materials.

### Linear mixed model analysis (BLUP)

To estimate the contributions of individual features to phenotypic variation, we implemented a linear mixed model framework based on BLUP [21, 41].

We have a feature matrix $\textbf{X} \in \mathbb {R}^{n \times p}$ and a response matrix $\textbf{Y} \in \mathbb {R}^{n \times q}$. Each omics layer was used to construct a corresponding kernel matrix $\textbf{K}_l$, which captures the pairwise similarities among samples. The univariate mixed model was performed for each phenotype $j = 1, \dots , q$ as follows:

$$ \begin{gathered} {\mathbf{y}}_{j} = {\mathbf{1}}\mu + \sum\limits_{{l = 1}}^{L} {{\mathbf{Z}}_{l} } {\mathbf{u}}_{{l,j}} + \boldsymbol{\epsilon} , \hfill \\ {\mathrm{Var}}[{\mathbf{y}}_{j} ] = \sum\limits_{{l = 1}}^{L} {{\mathbf{Z}}_{l} } {\mathbf{K}}_{l} {\mathbf{Z}}_{l}^{\prime } \sigma _{l}^{2} + {\mathbf{I}}\sigma _{e}^{2} . \hfill \\ \end{gathered} $$

The multi-kernel linear mixed effects model was computed using the R package RAINBOWR (version 0.1.35) [12]. The model was applied independently to each phenotype $j = 1, \dots , q$ to obtain trait-specific random effects $\textbf{u}_{l,j}$ for each omics layer *l*. Assuming that each kernel matrix was based on a linear inner product, we reconstructed the feature-level coefficients for each trait and omics layer using:

$$\begin{aligned} \textbf{K}_l = \frac{1}{p_l} \textbf{X}_l \textbf{X}_l^\prime , \quad \textbf{b}_{l,j} = \textbf{X}_l^\prime \textbf{K}_l^{-1} \textbf{u}_{l,j}, \end{aligned}$$

where $\textbf{X}_l \in \mathbb {R}^{n \times p_l}$ is each feature matrix (genome, metabolome, or microbiome), and $\textbf{b}_{l,j} \in \mathbb {R}^{p_l\times 1}$ is the resulting coefficient vector.

These feature matrices were used to construct the corresponding kernel matrices $\textbf{K}_l$ for every layer. All coefficient vectors were concatenated across the traits and layers to form a global coefficient matrix:

$$\begin{aligned} \textbf{B} = \left[ \textbf{b}_1, \dots , \textbf{b}_q \right] \in \mathbb {R}^{p \times q}, \quad \textbf{b}_j = \begin{bmatrix} \textbf{b}_{1j}\\ \vdots \\ \textbf{b}_{Lj} \end{bmatrix}\in \mathbb {R}^{p\times 1}. \end{aligned}$$

To enable comparison across traits, each column of $\textbf{B}$ was standardised to have a mean 0 and a variance 1:

$$\begin{aligned} \sum _{i=1}^{p} \textbf{B}_{i,j} = 0, \quad \frac{1}{p}\sum _{i=1}^{p} \textbf{B}_{i,j}^{2} = 1, \quad j = 1,\dots ,q. \end{aligned}$$

To examine the structure of the feature-trait relationships, we applied singular value decomposition (SVD) to the coefficient matrix:

$$\begin{aligned} \textbf{B} = \textbf{U} \textbf{D} \textbf{V}^\prime , \end{aligned}$$

where $\textbf{U}$ and $\textbf{V}$ are the left and right singular vectors, respectively, and $\textbf{D}$ is the diagonal matrix of singular values. The phenotypes and features were projected into the latent space using $\textbf{U} \sqrt{\textbf{D}}$ and $\textbf{V} \sqrt{\textbf{D}}$, respectively.

### Nonlinear feature importance using RF

We also implemented a nonlinear regression framework using RF to estimate feature contributions. The feature matrix $\textbf{X} \in \mathbb {R}^{n \times p}$ was constructed by concatenating multi-omics layers, while the response matrix $\textbf{Y} \in \mathbb {R}^{n \times q}$ represents the biomass-related phenotypic traits. Details of the data preprocessing are provided in Appendix A.

For each phenotype $j = 1, \dots , q$, we trained an independent RF model using the R package ranger (version 0.16.0) [44]. Hyperparameter tuning was performed using a grid search. See Appendix C and Fig. 7 for details. We confirmed that the predictive performance of the RF model fitted to the original data exceeded the null distribution of predictive performance obtained by refitting the model after randomly permuting the response vector (Fig. 8).

$$\begin{aligned} \textbf{y}_j = f_j(\textbf{X}) + \boldsymbol{\epsilon }_j, \end{aligned}$$

where $f_j$ denotes the RF model trained for phenotype *j*. After training, the feature importance scores were extracted. Let $\textbf{I} \in \mathbb {R}^{p \times q}$ denote the resulting matrix of feature contributions, where $\textbf{I}_{i,j}$ indicates the contribution of feature *i* to phenotype *j*.

The importance values from the RF were all positive. For the convenience of variable selection, each column of $\textbf{I} \in \mathbb {R}^{p \times q}$ is normalised such that the total sums to 1 for each phenotype:

$$\begin{aligned} \sum _{i=1}^{p} \textbf{I}_{i,j} = 1, \quad j = 1,\dots ,q. \end{aligned}$$

Analogous to the coefficient matrix $\textbf{B}$ derived from BLUP, the normalised importance matrix $\textbf{I} \in \mathbb {R}^{p \times q}$ from the RF models can be treated as a feature-by-trait matrix:

$$\begin{aligned} \mathbf {B^{\text {(RF)}}} = \textbf{I} \in \mathbb {R}^{p \times q}. \end{aligned}$$

Here, each column of $\mathbf {B^{\text {(RF)}}}$ represents the relative contribution of each feature to the prediction of a specific phenotype, aggregated across the trees in the forest (Fig. 1). While the entries of $\mathbf {B^{\text {(RF)}}}$ do not correspond to regression coefficients in a strict parametric sense, this matrix serves as a comparable construct for downstream analyses, such as dimensionality reduction or visualisation of feature-trait associations. Following the BLUP, we can perform the SVD for $\mathbf {B^{\text {(RF)}}}$ to visualise the structure of feature-trait relationships.

**Fig. 2:**
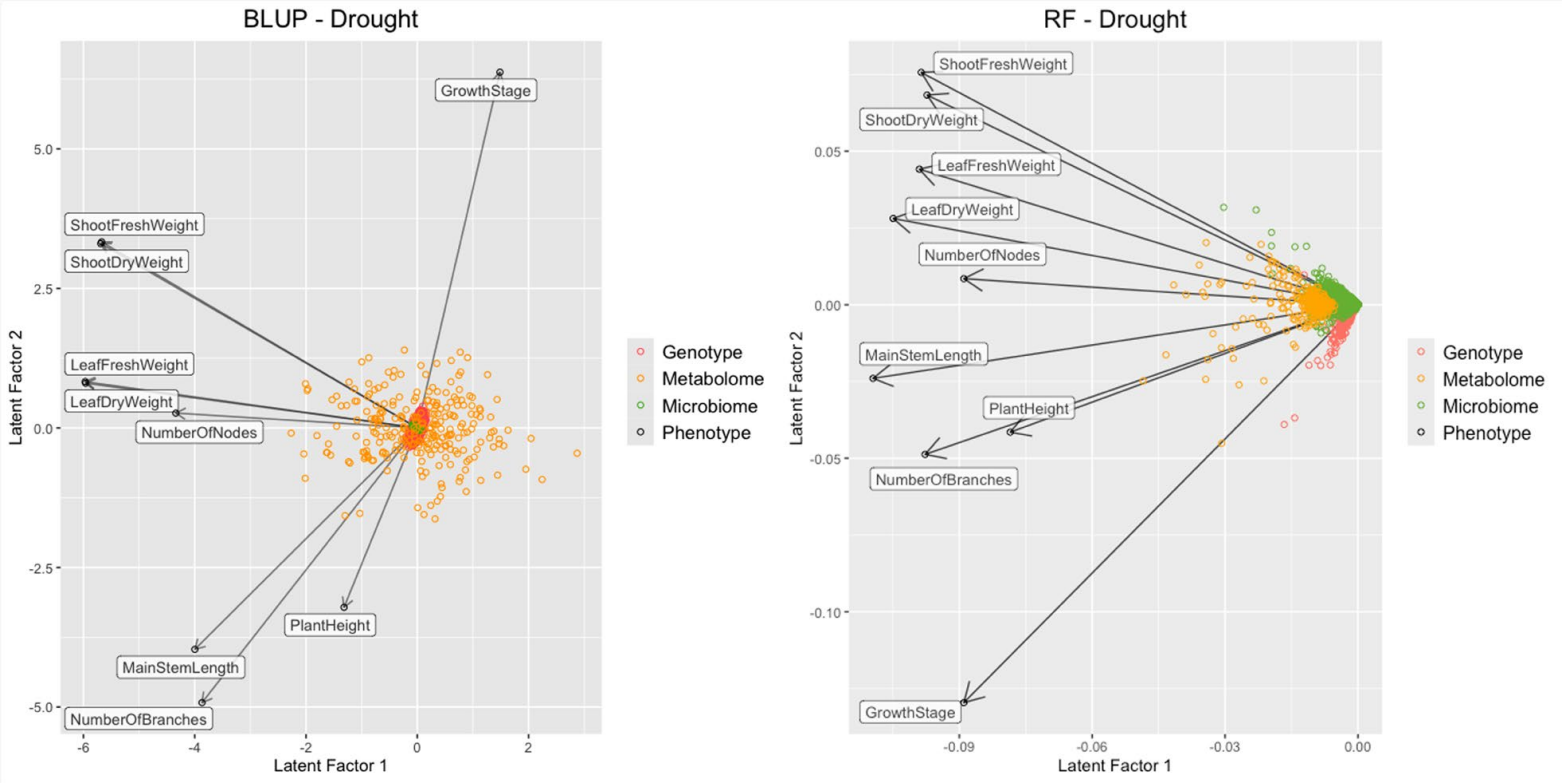
Feature–trait structures derived from BLUP and RF for predicting biomass-related phenotypes under drought conditions. Feature importance matrices from both models were subjected to singular value decomposition (SVD), and the first two latent factors are visualized. Points represent features and phenotypes projected into the latent space. Colors indicate data types (genotype, metabolome, or microbiome), and phenotype vectors are shown as arrows

Leaf dry weight, a commonly used indicator of plant biomass that reflects the size of the primary photosynthetic organ, showed representative predictability and consistent associations with omics features across all three omics layers (Fig. 2). Therefore, downstream GWAS and SHAP analyses were conducted using leaf dry weight as a representative phenotype for interpretability and computational feasibility.

### Genome-wide association study (GWAS)

GWAS is a linear approach for identifying SNP markers associated with phenotypic variation [3, 24]. Here, we employed GWAS in a conventional setting to evaluate the baseline performance and provide a comparison with SNP selection results from RF importance.

GWAS was performed using a linear mixed model (LMM) implemented in the R package gaston[26] (version1.6), incorporating three principal components (PCs) derived from the eigen decomposition of the GRM to account for population structure.

The full set of SNPs (425,858 markers, LD threshold = 0.95) was filtered using a minor allele frequency (MAF) threshold of 0.05 and then used in the GWAS model. After modeling, SNPs with a significance threshold of $p < 1 \times 10^{-5}$ were retained for further analysis.

### SHAP: individual-features attribution

To investigate the individual contributions of multi-omics features to phenotype prediction (hereafter referred to as individual feature contributions), we applied SHAP to the trained RF models. SHAP is a game-theoretic approach that quantifies the marginal contribution of each feature by comparing the model predictions across all possible subsets of the input features.

Formally, the SHAP value for feature *i* is defined as:

1 $$\begin{aligned} \phi _i(f, x) = \sum _{S \subseteq N \setminus \{i\}} \frac{|S|!\,(|N|-|S|-1)!}{|N|!} \left[ f_x(S \cup \{i\}) - f_x(S) \right] . \end{aligned}$$

where *N* is the set of all input features, *S* is a subset that excludes feature *i*, and *f* is the prediction model. The $f_x(S)$ denotes the model prediction for input instance *x* when only the features in *S* are considered. The left-hand side of Eq. (1) is a combination weight designed to give equal consideration to all possible feature orders. The right-hand side represents the marginal contribution to the prediction when feature *i* is added to subset *S*. By combining these components, feature importance can be assigned fairly, making SHAP particularly suitable for elucidating the nonlinear effects of features in multi-omics data.

SHAP values were computed for each sample using the iml R package (version 0.11.4) [23] and then averaged to obtain global feature importance scores.

After RF modeling under drought and control conditions separately, omics features were ranked based on their total variable importance scores, calculated for each feature $i$ as follows:

$$ \theta _i =\sum _{j=1}^{q} \mathbf {B^{\text {(RF)}}}_{i,j} $$

were retained, where $\mathbf {B^{\text {(RF)}}}_{i,j}$ denotes the contribution of omics feature $i$ to phenotype $j$.

Here, we selected features with a total RF importance score of $\theta _i > 0.01$ under either drought or control conditions, yielding a total of 159 features for evaluation. This threshold was chosen to retain features that exhibited non-negligible importance, given that the distribution of the feature importance scores was zero-inflated (Fig. 9). From a practical perspective, applying such a cutoff provides a reasonable preprocessing step for SHAP analysis because it reduces the computational burden while preserving the major informative signals.

Using this subset of 159 features, the SHAP values were calculated to identify environment-dependent differences in the relative contribution of individual features. For each condition, the absolute SHAP values were first calculated for each feature across all samples and then averaged. The ratio of the mean absolute SHAP values (drought divided by control) was calculated to highlight the condition-specific features of the model.

### SHAP: interaction-features attribution

#### Condition specific interactions

Although SHAP individual-feature attribution values quantify each omics feature, they do not capture interaction effects. To address this, we applied SHAP interaction analysis to characterise the pairwise relationships among the features [22].

SHAP interaction values decompose the prediction into individual effects and pairwise interactions.

2 $$\begin{aligned} \phi _{i,j}(f,x) = \sum _{S \subseteq N \setminus \{i,j\}} \frac{|S|!\,(N-|S|-2)!}{2(N-1)!} \, \delta _{ij}(S), \end{aligned}$$

where $$i \ne j$$ and

3 $$\begin{aligned} \delta _{ij}(S) = f_x(S \cup \{i,j\}) - f_x(S \cup \{i\}) - f_x(S \cup \{j\}) + f_x(S). \end{aligned}$$

The weighting factor in Eq. (2) ensures the symmetry consideration of all feature orderings, whereas the term $\delta _{ij}(S)$ isolates the second-order marginal contribution in Eq. (3).

Although some features exhibited only minor individual effects on the phenotype, they might contribute to phenotypic variation through cooperative or context-dependent interactions with other omics layers. To capture such synergy-driven patterns, we adopted a broader feature set ($\theta _i > 0.001$ in both conditions), which facilitated the identification of interaction effects that were not reflected in the individual feature importance. The distribution of feature importance values is shown in Fig. 9.

To evaluate the sensitivity of the inferred interaction network to the RF importance threshold $\theta$, we conducted a robustness analysis by varying $\theta$ from 0.001 to 0.01. SHAP interaction scores were first computed under the most permissive setting ($\theta = 0.001$). For each stricter threshold, nodes retained according to RF importance were identified for each condition, and the interaction network was restricted to edges whose two incident nodes remained selected.

This yielded a total of 2729 features for the evaluation. Based on these features, we constructed omics feature interaction networks, where nodes represent individual features and edges denote the strength of interaction between feature pairs.

In high-dimensional SHAP interaction analysis, the number of feature pairs increases exponentially with the number of input variables. Due to this computational burden, the interaction values were computed using the shap.TreeExplainer from the shap Python package applied to an XGBoost-based gradient boosting model [6], as RF-based implementations were computationally prohibitive in this setting. Since both RF and gradient boosting are tree-based ensemble methods, the resulting SHAP interaction values captured the same class of nonlinear feature interactions underlying the nonlinear prediction models.

Hyperparameter tuning was conducted so that the same set of tuned hyperparameters could be applied to both the control and drought conditions, ensuring comparability between environmental settings. See Appendix C and Fig. 7 for implementation details.

SHAP interaction values were calculated for each sample and averaged to obtain the global $2729 \times 2729$ symmetrical interaction matrices for each condition. To highlight the environment-specific interactions, we computed a differential SHAP interaction matrix as:

$$ \Delta \boldsymbol{\Phi } = \boldsymbol{\Phi }_{\text {drought}} - \boldsymbol{\Phi }_{\text {control}}, $$

where $\boldsymbol{\Phi }$ denotes a SHAP-based pairwise interaction matrix. Positive values of $\Delta \boldsymbol{\Phi }$ indicated stronger interactions under drought stress, whereas negative values indicated interactions that were more pronounced under control conditions.

The two-step SHAP analysis–first focusing on individual feature importance, then exploring pairwise interaction features–provides a comprehensive and interpretable view of how genomic, metabolomic, and microbial features jointly influence plant phenotypes under contrasting environmental conditions.

#### Null-distribution-based significance assessment

To assess the statistical significance of each differential interaction, a permutation-based null distribution was constructed. For each condition, the response vector was randomly permuted. The XGBoost model was then refitted to the permuted data, and SHAP interaction matrices were computed. For each permutation replicate $b \in \{1,\ldots ,100\}$, a null differential interaction matrix was calculated as:

$$ \Delta \boldsymbol{\Phi }^{(b)} = \boldsymbol{\Phi }^{(b)}_{\text {drought}} - \boldsymbol{\Phi }^{(b)}_{\text {control}}, $$

representing the distribution of interaction differences expected under the null hypothesis of no association between predictors and observed values (phenotypes).

All off-diagonal (upper-triangular) entries from the $B=100$ null differential interaction matrices were concatenated to form a pooled null distribution, from which the mean $\mu _{0}$ and standard deviation $\sigma _{0}$ were estimated.

The observed differential interaction values were converted into Z-scores as:

$$ Z_{ij} = \frac{\Delta \Phi _{ij} - \mu _{0}}{\sigma _{0}}, $$

Here, edges with large absolute Z-scores were interpreted as highly environment-specific SHAP interactions.

Edgewise empirical *p* values were computed for each feature pair (*i*, *j*) using two-sided tests based on the null replicates [28].

$$ p_{ij} = \frac{ 1 + \sum _{b=1}^{B} \mathbb {I}\!\left( \left| \Delta \Phi ^{(b)}_{ij}\right| \ge \left| \Delta \Phi _{ij}\right| \right) }{ B + 1 }, $$

To examine the robustness of the inferred interaction network with respect to the choice of the Z-score threshold, we systematically constructed networks using a range of alternative cutoffs ($|Z| \ge 10, 20, 30, 40, 50,$ and 60). For each threshold, the corresponding two-sided empirical *p* values were jointly evaluated to ensure consistency between effect size and statistical extremeness.

Although the overall qualitative network structure was preserved across the thresholds, a cutoff of $|Z| \ge 30$ was selected for the downstream analyses as a pragmatic compromise between statistical stringency and network interpretability. This threshold corresponds to the extreme tail of the pooled null distribution of SHAP interaction differences (Fig. 10) and effectively filters out interactions that are likely attributable to resampling noise. Under this cutoff, the resulting network comprised 160 connections, representing only 0.004% of all possible feature pairs, yielding a sparse yet interpretable representation of robust environment-specific interactions.

#### Stability analysis of interaction edges using bootstrap resampling

Because SHAP interaction matrices are estimated from finite samples, the individual edge differences in $\Delta \boldsymbol{\Phi }$ may be sensitive to resampling variability. To assess the robustness of the differential interactions, edge-level stability was quantified using an additional bootstrap procedure applied to the observed data.

Specifically, for each condition, we generated 100 bootstrap replicates by resampling individuals with replacements and refitting the XGBoost model on the resampled pairs $\{(\textbf{x}_{k}, y_{k})\}$. For each replicate *b*, we computed the bootstrap differential interaction matrix

$$ \Delta \boldsymbol{\Phi }^{(b)}_{\textrm{boot}} = \boldsymbol{\Phi }^{(b)}_{\textrm{drought,boot}} - \boldsymbol{\Phi }^{(b)}_{\textrm{control,boot}} $$

Using these replicates, the complementary stability measures for each edge were defined, (*i*, *j*). The presence stability was defined as the number of bootstrap replicates for which a given edge (*i*, *j*) is observed as nonzero.

$$ n^{\textrm{obs}}_{ij} = \sum _{b=1}^{B_{\textrm{boot}}} \mathbb {I}\!\left( \left| \Delta \Phi ^{(b)}_{\textrm{boot},ij}\right| > \varepsilon \right) , $$

where $\varepsilon =10^{-12}$ is the small numerical tolerance. This measure aims to evaluate the reproducibility of the interaction under resampling, which is particularly relevant for sparse or zero-inflated interaction matrices.

To suppress the spurious edges driven by resampling noise, a stability-based filtering criterion was applied, and edges with $n^{\textrm{obs}}_{ij} < 2$ were excluded from downstream network analyses. Given the extreme sparsity of SHAP interaction matrices ($\approx 0.015\%$, corresponding to approximately 500 of 3.7 million possible pairs), the appearance of a purely spurious edge in each bootstrap replicate could be treated as a rare event. Under a simple binomial model $X \sim \textrm{Binomial}(B_{\textrm{boot}}=100, p)$ with $p \approx 1.5 \times 10^{-4}$, the probability that the edge observed in at least two bootstrap replicates is approximately $1.1 \times 10^{-4}$. See Appendix "Stability-based filtering of differential interactions" for further statistical justification for this conservative threshold. Thus, the stability metric: $n^{\textrm{obs}}_{ij}$ is reported alongside the Z-scores and empirical *p* values to facilitate interpretation of the edge-level robustness.

## Results

### RF and BLUP models

Figure 2 presents the SVD of the coefficient matrix of BLUP and the importance matrix of RF for the drought conditions. The results demonstrate that the RF model exhibited a more pronounced and variable pattern of feature importance than the BLUP model. Notably, in BLUP, metabolites are the features most correlated with phenotypes, while other features (such as the genome and microbiome) exhibit a shrinkage pattern, indicating that metabolome features primarily contribute to phenotypic prediction. Under control conditions, a similar trend was observed (Fig. 11).

### SHAP analysis of individual-features

SHAP analysis suggests key condition-specific features contributing to phenotypic variations under drought and control conditions. Since metabolomic and microbiomic profiling do not cover the entirety of omics information, the highlighted features should be understood as those selected from the analysed subset described in the Materials and Methods.

Under drought stress, the most highly contributing features were predominantly flavonoids, including isoflavones (e.g. daidzin and its derivatives and genistin) and flavone/flavanone glucosides (e.g. apigenin-7-O-glucoside and naringenin-7-O-glucoside). Flavonoids play a major role in plant responses to drought stress and are associated with plant-microbe interactions [8, 14]. Additionally, we identified amino acids and their derivatives (asparagine, ornithine, and indole-3-carboxyaldehyde) as major contributing features.

Furthermore, *Candidatus Nitrosocosmicus*, a clade of ammonia-oxidising archaea (AOA), was particularly selected under drought. This clade is notable for its ability to produce manganese catalase (MnKat), which decomposes hydrogen peroxide (H_2_O_2_), a major reactive oxygen species (ROS) [32]. This enzymatic capacity enables *Ca. Nitrosocosmicus* to survive in oxidative environments, such as drought-stressed soils. Its dominance under ROS stress conditions highlights its potential role in AOA-plant interactions [17], consistent with our findings.

In contrast, under control conditions, genetic markers contributed significantly to the observed traits. SHAP re-identified the SNP Chr06-15056895 as an important feature, which was also detected by GWAS (Fig. 12). This SNP encodes a copper amine oxidase family protein involved in amine metabolism, tissue differentiation, and organ development in plants [36]. An SNP Chr04-22035062, located near the gene *Glyma.04G140400*, was also selected. This gene encodes an oxidoreductase enzyme that is potentially involved in plant growth [9]. Another notable marker, SNP Chr04-16278976, is located near *Glyma.04G127100* [40]. This gene encodes a receptor-like kinase that contains both a protein kinase-like domain (IPR011009) and a Gnk2-homologous domain (IPR002902) and is involved in signal transduction pathways [34].

In addition to genetic factors, *Duganella* was identified as an important microbial contributor under control conditions in this study. Several species of *Duganella* are known for their plant growth-promoting properties, including the solubilisation of essential nutrients, such as phosphorus, potassium, and zinc [42].

Some metabolites, such as gamma-aminobutyric acid (GABA), were selected in both control and drought conditions; however, they were not classified as condition-specific factors because their levels were similarly elevated under both conditions. Further details of each condition are presented in Fig. 13.

### SHAP analysis of interaction-features

SHAP interaction values enabled a detailed investigation of multi-omics feature interactions. Condition-specific connectivity patterns were identified by comparing network differences between the drought and control conditions using a Z-score threshold of $|Z| \ge 30$ (Fig. 10). Under this threshold, major features were identified based on their connectivity with other features, including GABA and daidzin, both of which are known to promote plant growth and to influence rhizosphere microbiome composition, particularly under drought stress [4, 8, 30, 46, 49]. The corresponding networks were obtained by using alternative Z-score thresholds ($|Z| \ge 10,20, 30, 40, 50, 60$), and are shown in Fig. 14.

Several of these interactions correspond to the known biological relationships. Notably, the model identified a strong interaction between daidzin and the bacterium *Paenibacillus* under drought conditions. This may be supported by a previous study showing that certain strains of *Paenibacillus* produce $\beta$-glucosidases that catalyse the hydrolysis of glycosidic bonds in isoflavone glucosides, such as daidzin, thereby converting them into their active aglycone forms, such as daidzein [25]. In addition, the model suggested a biologically plausible interaction between *Paenibacillus* and GABA under drought conditions. This aligns with reports that *Paenibacillus* can express glutamate decarboxylase, facilitating the conversion of glutamate to GABA [35].

Under control conditions, a notable interaction was observed involving SNP Chr04-22035062, which was also selected in the individual-feature analysis, located downstream of the gene *Glyma.04G140400* (oxidoreductase enzyme), potentially involved in metabolic responses to GABA.

We additionally evaluated the sensitivity of the inferred interaction network to the RF importance threshold $\theta$. The main biological interactions reported in the network remained stable across a wide range of $\theta$ values (Table 1). For example, the interaction between *Paenibacillus* and daidzin was retained for $0.001 \le \theta \le 0.003$, the interaction between *Paenibacillus* and GABA for $0.001 \le \theta \le 0.006$, the interaction between Chr04_22035062 and GABA for $0.001 \le \theta \le 0.01$, and the interaction between *Rhodanobacter* and daidzin for $0.001 \le \theta \le 0.005$. These results confirm that the principal interaction structure was robust to moderate changes in the preprocessing threshold (Fig. 15).

To assess the robustness of the detected interactions, their stability across 100 bootstrap replicates was evaluated (Tables 2 and 3). Among bootstrap replicates, several edges showed moderate stability. The edge between SNP Chr04-22035062 and GABA exhibited relatively higher stability (observed in 14 bootstrap replicates). The interactions between *Paenibacillus* and daidzin (6 replicates) and between *Paenibacillus* and GABA (3 replicates) appeared across resampling iterations. Similarly, an interaction between *Rhodanobacter* and daidzin was detected in 6 replicates, suggesting a reproducible association between the taxon and isoflavone-related metabolites. However, the magnitude of SHAP interaction differences (Drought − Control) was not necessarily proportional to the replicative stability (Fig.  16).

## Discussion

The SVD of the feature-trait matrices for BLUP and RF showed a more dispersed and interpretable structure for the RF-based models, particularly under drought conditions (Fig. 2 and  11). In contrast, BLUP showed more shrinking effects, which is typical when the explanatory matrix contains many variables. This suggests that a nonlinear model may be better suited for feature selection in complex biological systems, where additive linear assumptions do not hold.

The overlap between SNPs identified by GWAS and those selected by SHAP highlights the potential synergy between traditional and machine learning-based approaches (Fig. 12). For instance, SNP Chr06-15056895, located near the gene *Glyma.06G178400*, was detected by both SHAP and GWAS, reinforcing its putative role in growth-related traits. Such convergence enhances confidence in candidate biomarkers.

**Fig. 3:**
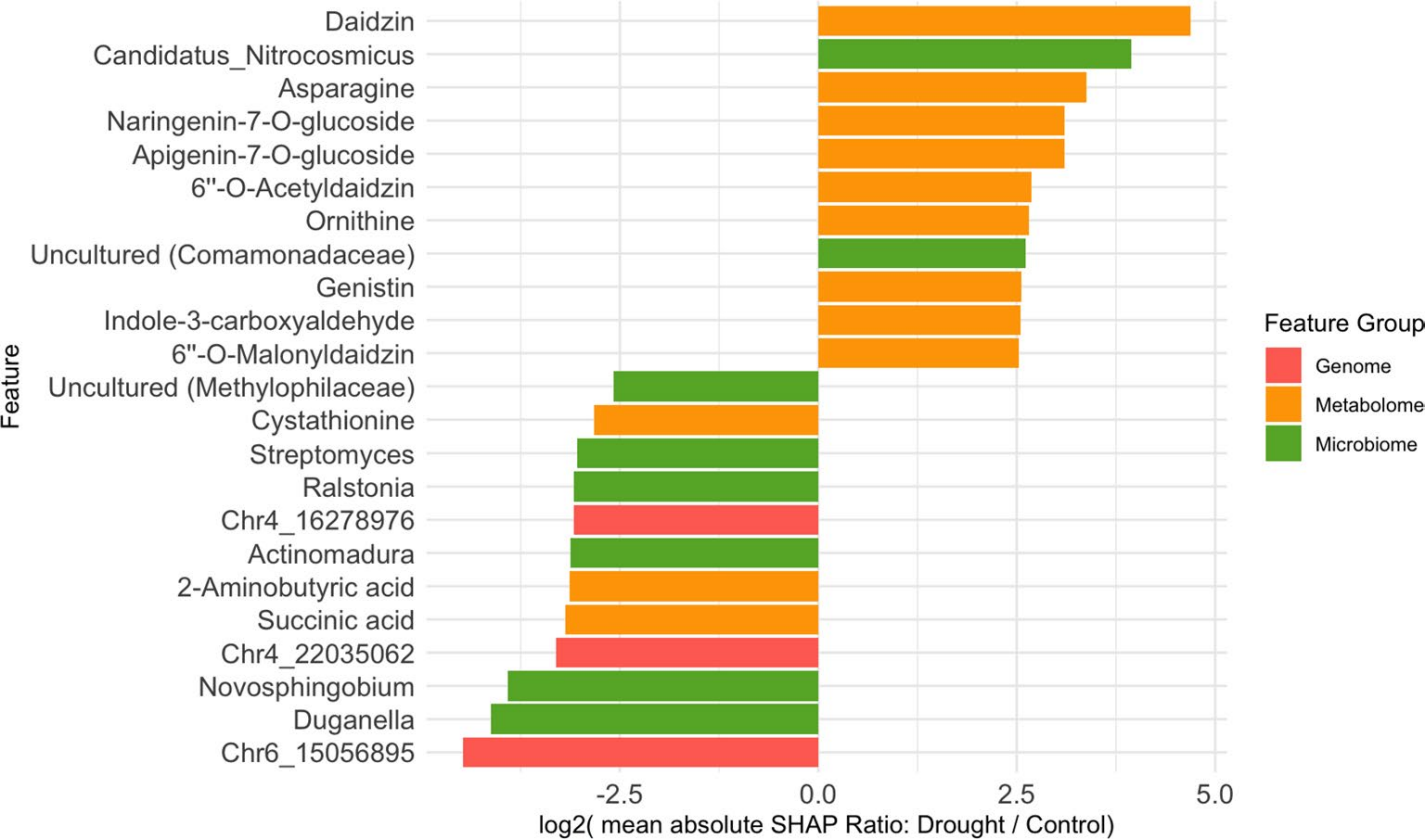
SHAP-based individual feature importance. For each control and drought condition, absolute SHAP values were computed and their ratios were calculated. The bar plot shows the $\log _{2}$ ratio of the mean absolute SHAP values (drought divided by control). Features with $\log _{2}(\text {mean absolute SHAP ratio}) > 2.5$ are displayed. Positive values indicate features more specific to drought conditions, whereas negative values indicate features more specific to control conditions

Our findings suggest a shift in the dominant omics layers under drought stress, highlighting the enhanced roles of metabolomic and microbial features (Fig. 3). Notably, the strong contribution of flavonoids, including daidzin, supports previous evidence that soybean roots adjust flavonoid exudation to shape the rhizosphere community in response to drought stress [8, 14]. The identification of amino acids and their derivatives is also consistent with previous findings, as amino acid concentrations increase under drought conditions [29]. Additionally, the selection of *Candidatus Nitrosocosmicus*, a major contributor to phenotypic variation under drought stress, further validates the SHAP model’s identification of key features in the microbial community [17, 32].

**Fig. 4:**
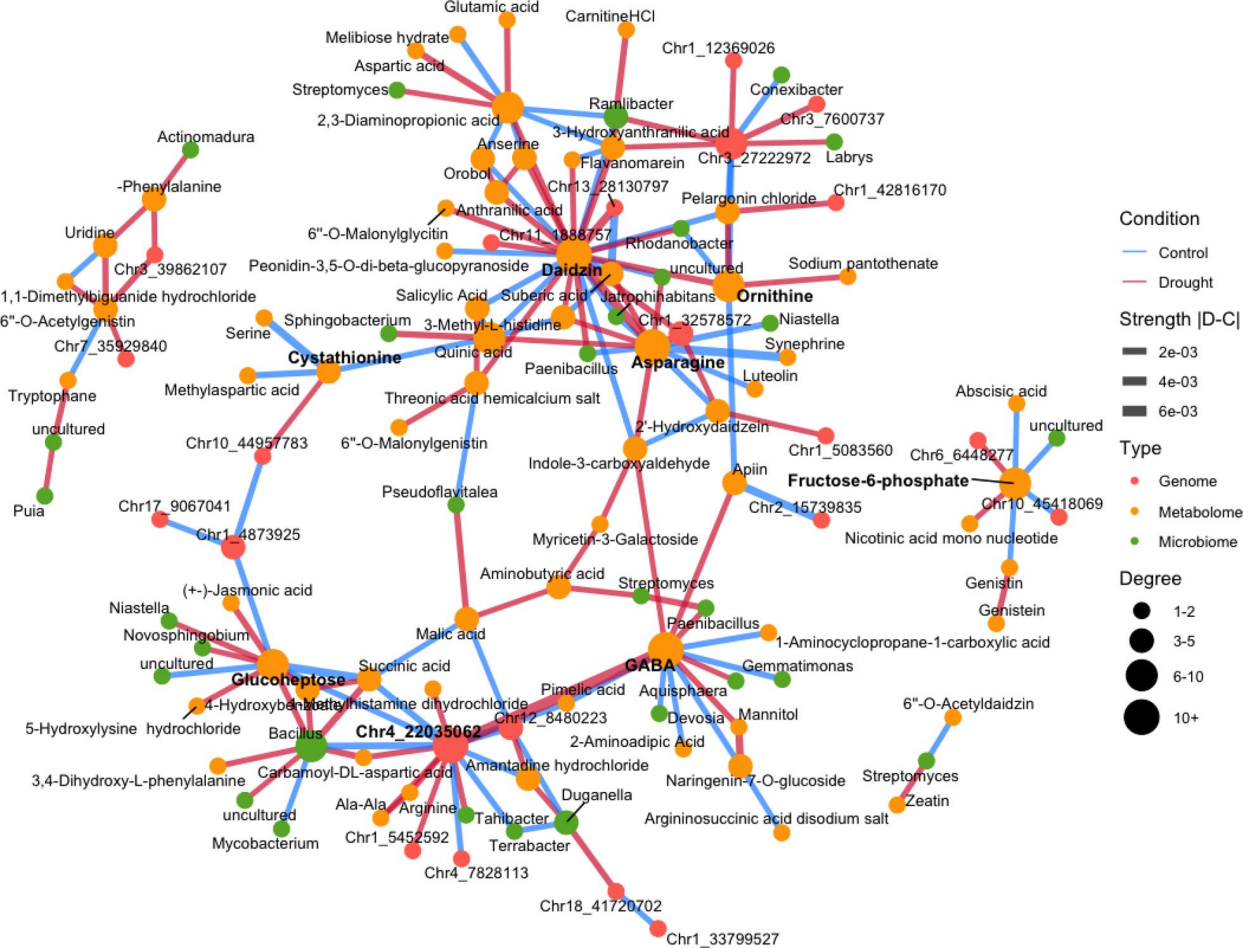
Condition-specific SHAP interaction difference network. The model was trained separately to predict biomass (leaf dry weight) under drought and control conditions. Environment-specific interactions were identified from the differential SHAP interaction matrix (drought minus control) using Z-scores derived from a pooled null distribution. Only interactions with large absolute Z-scores ($|Z| \ge 30$) were retained. Red edges represent interactions that are stronger under drought conditions, whereas blue edges indicate interactions that are more pronounced under control conditions. Node colors denote omics layers: genome SNP markers (red), microbiome features (green), and metabolome features (orange). Node size reflects the number of connections (degree), edge width corresponds to the magnitude of the interaction difference between drought and control, and highly connected features (hubs) are highlighted in bold font

**Fig. 5:**
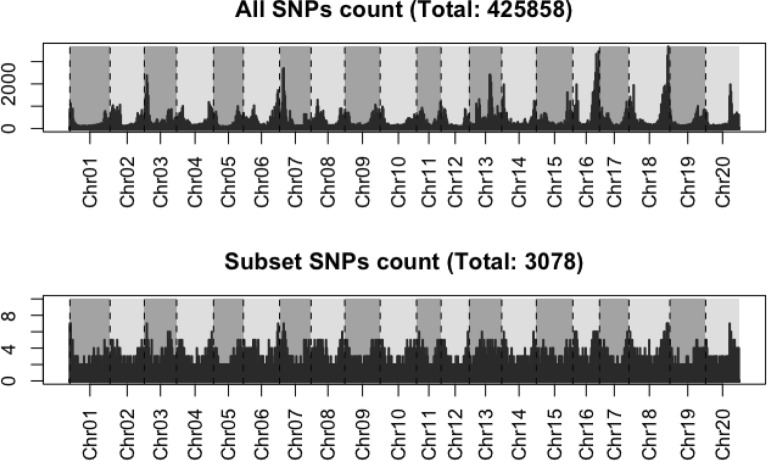
SNPs coverage comparison. The SNP mapping in the full set of 425,838 SNPs ( LD threshold of 0.95) and a subset of 3078 SNPs filtered by a LD threshold of 0.001

**Fig. 6:**
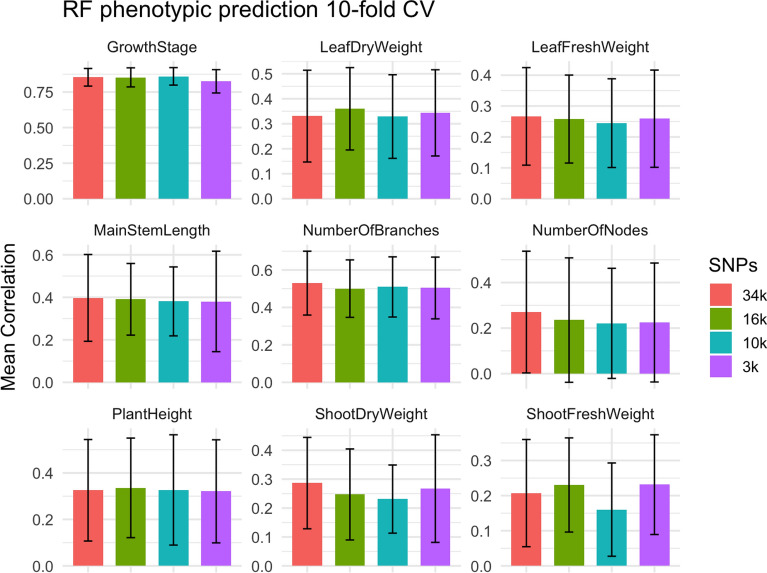
Mean Pearson correlation (±1 SD) from 10-fold cross-validation of RF models predicting phenotypes. Bars compare SNP marker sets of different sizes; 34k (34,632), 16k(16,419), 10k(10,143), and 3k(3,078) loci, respectively. The legend shows the number of markers

**Fig. 7:**
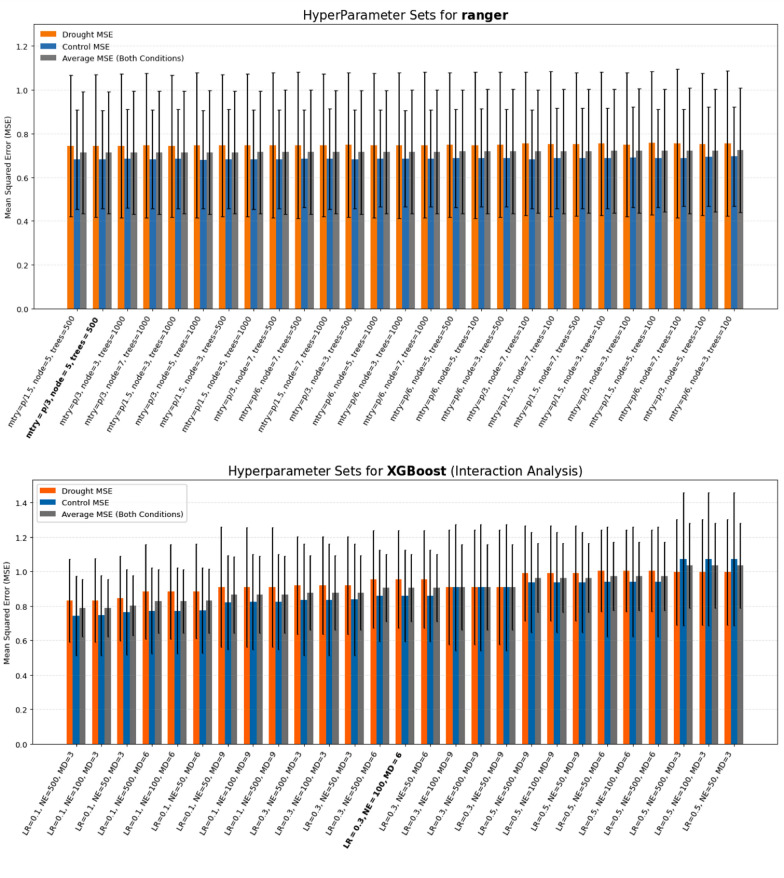
Hyperparameter tuning for phenotypic prediction under drought and control conditions. Top: Ranger, Bottom: XGBoost. Each bar represents the mean squared error (MSE) averaged over 10-fold cross-validation, with error bars indicating the standard deviation across folds for a specific combination of three hyperparameters. Orange bars represent MSE under drought conditions, blue bars represent MSE under control conditions, and gray bars show the average MSE across both conditions. The hyperparameter combinations selected for this study are highlighted in bold

**Fig. 8:**
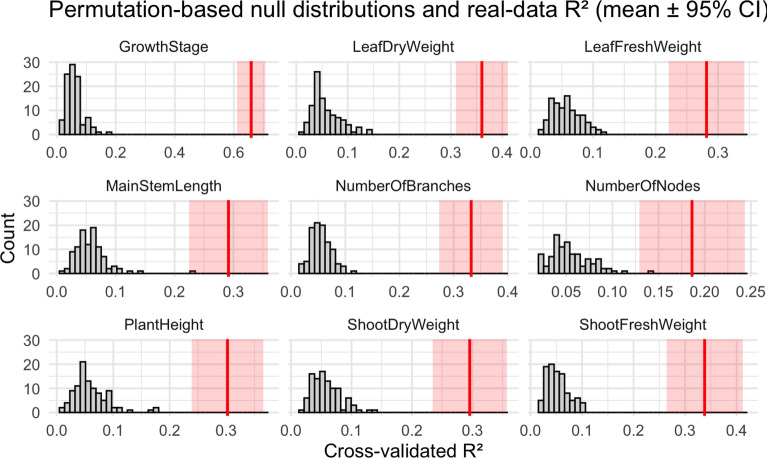
Null distribution of the cross-validated coefficient of determination ($R^2$) obtained by permuting the phenotypic labels (100 permutations, 10-fold cross-validation each) in phenotypic prediction by RF model (input: genome, microbiome, and metabolome). The gray histogram represents the null distribution under the assumption of no association between predictors and phenotype. The vertical red line indicates the mean $R^2$ obtained from repeated 5-fold cross-validation using the real data, and the shaded region denotes its 95% confidence interval (mean ± 1.96 SE). The real-data $R^2$ lies far outside the null distribution, demonstrating that the predictive performance cannot be explained by random associations

**Fig. 9:**
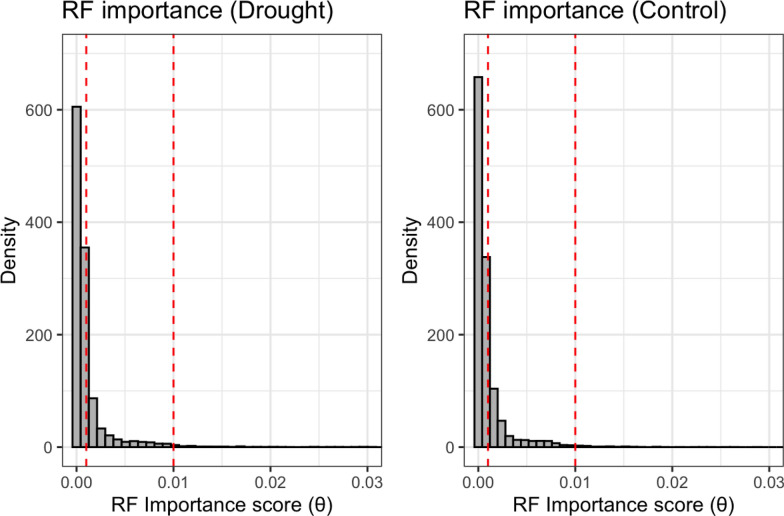
Distribution of RF importance value $\theta$. The vertical lines show the threshold ($\theta$=0.001,0.01). Approximately 1% and 25% of features are retained at these thresholds, respectively

**Fig. 10:**
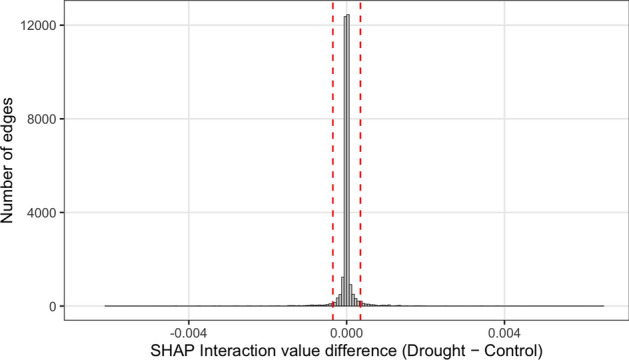
Distribution of SHAP interaction value differences (Drought – Control) across all feature pairs. Only non-zero interaction edges are shown (5,563 of 3,716,793). Red dashed vertical lines indicate the thresholds corresponding to $Z = \pm 30$, derived from the pooled null distribution

**Fig. 11:**
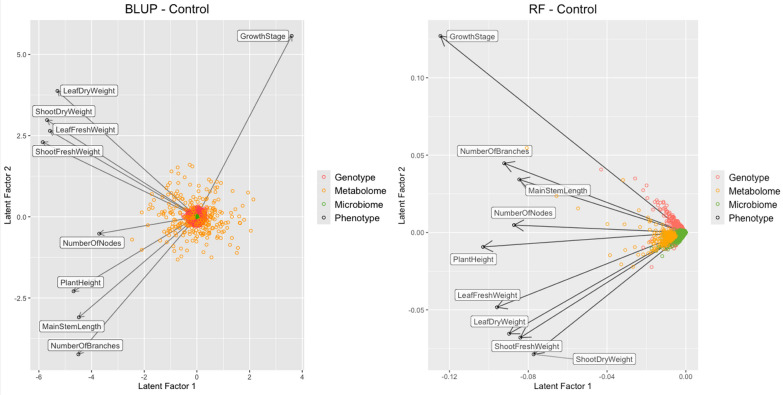
Feature-trait structures derived from BLUP and RF in predicting biomass-related phenotypes under control conditions. Feature importance matrices from both models were subjected to singular value decomposition (SVD), and the first two latent factors are visualized. Points represent features and phenotypes projected into the latent space. Color indicates data type: genotype, metabolome, or microbiome. Vectors for phenotypes are shown as arrows

**Fig. 12:**
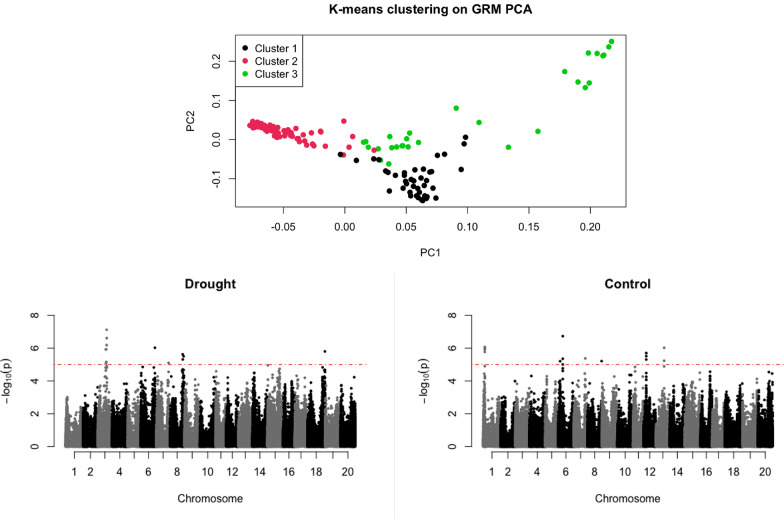
The genomic clustering and GWAS results. Top plot: PCA (principal component analysis) and k-means clustering of genomic relationship matrix (covariance matrix of SNP markers). Bottom plot: Results of GWAS. The red horizontal dotted line indicates the threshold of $p < 1 \times 10^{-5}$. GWAS was performed by incorporating the three principal components

**Fig. 13:**
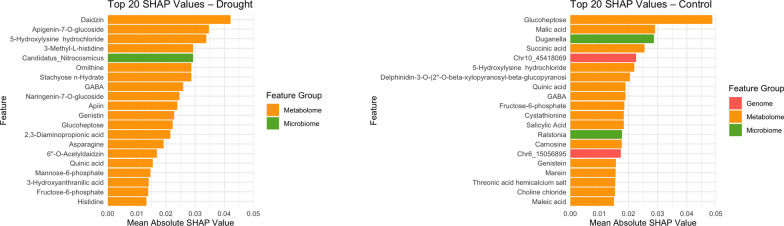
Top 20 SHAP values in drought and control conditions

**Fig. 14:**
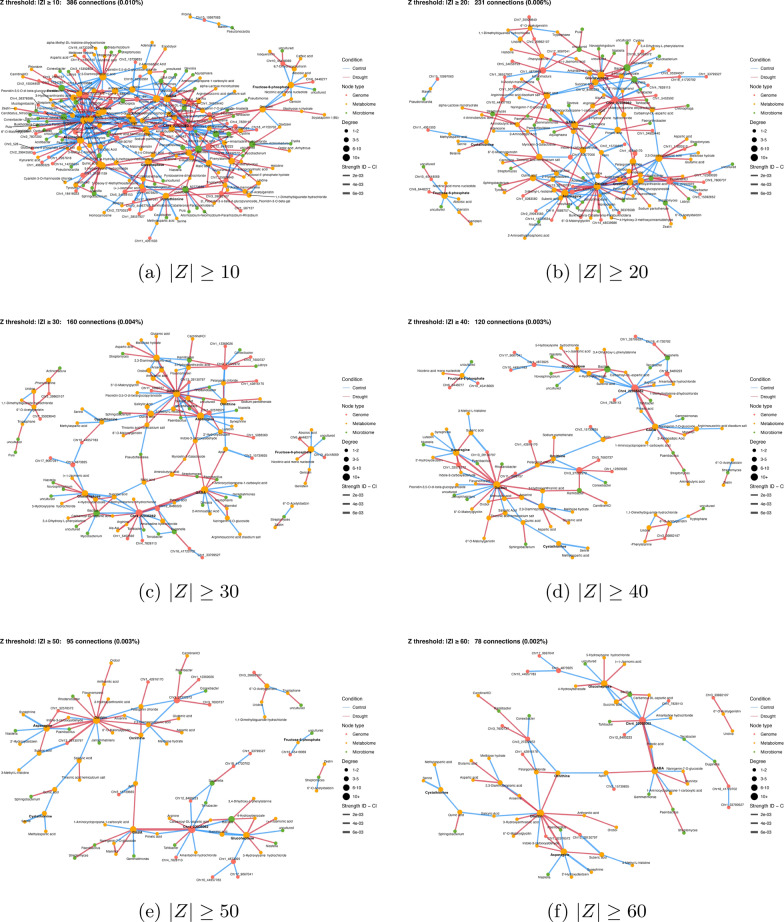
Condition-specific SHAP interaction difference networks under varying Z-score thresholds. Networks were constructed from the differential SHAP interaction matrix (drought minus control), retaining only edges with large absolute Z-scores. Increasing the Z threshold progressively restricts the network to more extreme, environment-specific interactions

**Fig. 15:**
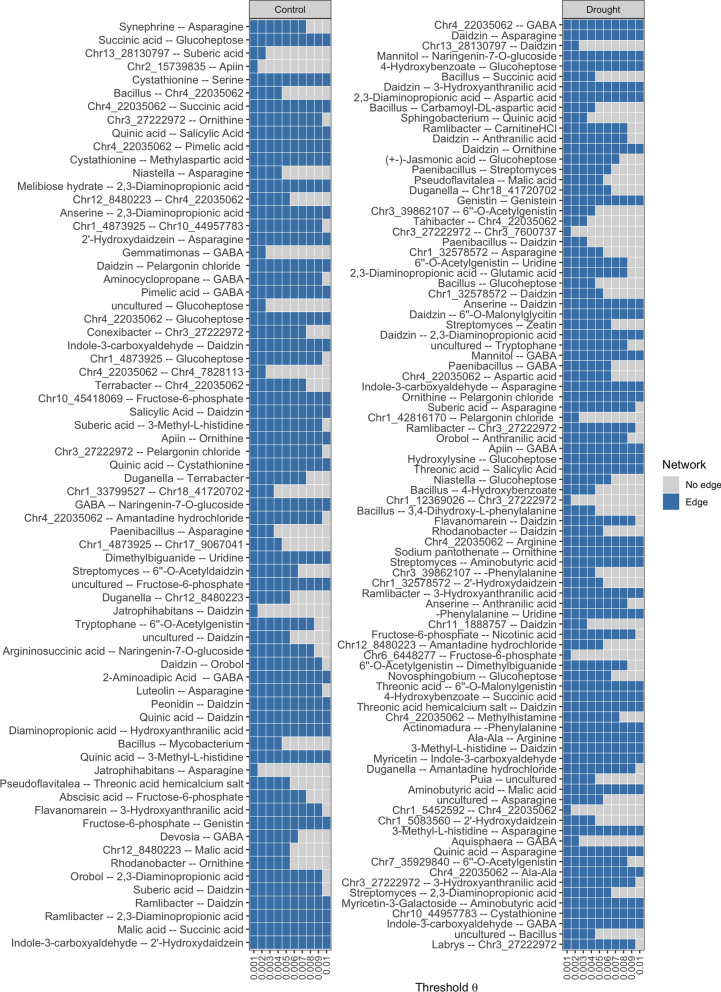
Sensitivity analysis of the SHAP interaction network to the Random Forest importance threshold $\theta$. The SHAP interaction network was first constructed under the most permissive threshold ($\theta = 0.001$), and the retained edge set was then re-evaluated by restricting the network to nodes that remained selected under increasingly stringent $\theta$ values. For each threshold, an edge was retained only when both of its incident nodes were still included after RF-based feature filtering. The two panels show drought-specific and control-specific subnetworks, respectively, and each row corresponds to one previously identified edge with $|Z| \ge 30$

Interaction analysis provided further plausible plant-metabolic-microbial links (Fig. 4). For example, daidzin, which was selected as an individual feature, was also selected as a feature of the pairwise interactions. Especially, the co-selection of daidzin and *Paenibacillus* aligns with reports that *Paenibacillus* strains produce $\beta$-glucosidases to hydrolyse isoflavone glucosides such as daidzin [25]. This enzymatic synergy may be related to ROS management. Additionally, the inferred interaction between *Paenibacillus* and GABA agrees with evidence that this bacterium encodes glutamate decarboxylase [35], which could support plant stress resilience. Interestingly, although GABA was not selected as a condition-specific feature based on individual SHAP contributions, network-level analysis revealed a strong association with drought-specific activity. This highlights the advantage of network approaches in capturing complex indirect relationships that are not apparent from individual feature contributions alone.

In addition to these known interactions, the model also suggests other potential connections between plant secondary metabolites and the microbiome that have not yet been directly reported. For example, daidzin is associated with bacteria such as *Rhodanobacter* [35], which is known to promote plant growth. GABA was also connected with *Devosia*, a plant growth–promoting genus [48], and *Aquisphaera*, which is frequently detected in the plant rhizosphere [37]. These findings highlight the potential to elucidate previously unknown plant-microbe interactions, in which plants may use flavonoids and other metabolome features to modulate the composition of rhizosphere bacteria [8, 13, 14].

Interestingly, our analysis showed contrasting patterns of feature associations under drought and control conditions. Under drought stress, metabolomic features were particularly prominent in the modeling, suggesting scenarios in which multiple metabolites are simultaneously produced by plants to cope with drought stress. For instance, the correlation between ornithine and asparagine may reflect amino acid accumulation patterns that influence plant stress responses [29]. This highlights the importance of metabolite co-regulation and network-level interactions during abiotic stress.

In contrast, under control (non-drought) conditions, genetic determinants were more strongly associated with trait expression, reflecting the predominance of intrinsic growth programs and host-regulated physiological processes in the absence of stress, that is, genetic effects outweighing environmental influences. This is supported by the identification of genotypic markers near genes involved in signal transduction, amine metabolism, and development.

Interaction edges were observed in only a limited number of bootstrap replicates; however, this pattern is a natural consequence of the extremely high-dimensional and sparse interaction spaces in which only 0.015% of all possible feature pairs exhibited non-zero SHAP interactions. A simple binomial argument indicates that observing the same interaction in as few as 2–4 of 100 bootstrap replicates is highly unlikely under a null model driven purely by sparsity. Given the empirical nonzero rate of SHAP interactions ($p \approx 1.5 \times 10^{-4}$), the probability of observing an edge at least twice, three times, or four times by chance alone is of the order of $10^{-4}$, $10^{-7}$, and $10^{-9}$, respectively. Thus, even modest levels of replicative stability can provide non-trivial statistical support in this setting (Appendix "Stability-based filtering of differential interactions").

**Fig. 16:**
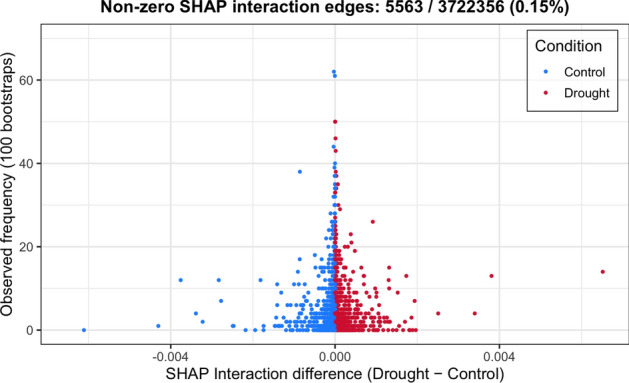
Stability of SHAP interaction differences between drought and control. Each point represents one feature–feature interaction edge. The x-axis shows the signed difference in SHAP interaction strength (Drought − Control) from the point estimate, and the y-axis shows the number of bootstrap replicates (out of 100) in which the edge was observed as non-zero (5563/3722356; 0.15 %). Colors indicate the direction of the point-estimate difference (positive: drought-enriched; negative: control-enriched)

Some interactions exhibit large point-estimate differences but limited bootstrap recurrence, whereas others show moderate differences with higher stability (Fig. 16). This pattern may partly arise from the severe multicollinearity inherent in high-dimensional data such as those analyzed in this study, which complicates feature selection. In such settings, the model may alternate between correlated features across bootstrap samples, leading to reduced apparent stability. This issue can potentially be mitigated by incorporating stronger regularization or dimension-reduction approaches.

In the present study, we intentionally prioritized the detection and biological interpretation of interaction effects, using stability as a supportive criterion. Alternative analytical designs are also feasible. For example, greater emphasis could be placed on stability by adjusting the model complexity, regularization strength, or low-dimensional feature selection thresholds to enhance the collective recurrence of interactions. Such strategies would likely enable analyses to focus more explicitly on reproducibility.

Overall, the integrated framework enables scalable feature selection across multiple omics layers, including plant genomic data, and highlights potentially meaningful biological interactions. Despite this choice, there were some limitations. Although the proposed framework identifies synergistic associations across omics layers, it does not establish causal relationships between features. Therefore, the resulting interaction network reflects model-based associated contributions rather than direct mechanistic links, and should be interpreted as a hypothesis-generating representation to guide targeted validation.

Accordingly, further experimental studies are required to validate and extend our findings. Causal biological mechanisms can be explored through targeted experimental designs, such as quantitative trait locus (QTL) mapping and gene expression analyses, which will help refine candidate loci or genes and elucidate the underlying gene regulatory networks.

Moreover, we plan to expand this framework to incorporate larger untargeted metabolomics and microbiome datasets spanning a broader range of organisms, including fungi. Such extensions will facilitate investigations of broader ecological and ecosystem-level processes and provide deeper insights into the complex dynamics that shape these systems.

## Conclusions

This study demonstrates the utility of comparing linear and nonlinear modeling approaches to validate feature selection in multi-omics datasets. By comparing BLUP and GWAS with a nonlinear machine learning model across genomic, metabolomic, and microbiomic layers, we confirmed that the RF model captured additional nonlinear interactions, avoiding the shrinkage effects observed in linear methods. The use of SHAP further ensured that these nonlinear patterns were biologically interpretable and not model artefacts. We identified environment-specific biomarkers linked to soybean biomass under drought and control conditions, providing a basis for predictive breeding and microbiome-informed strategies to improve stress resilience. Future studies incorporating temporal sampling and experimental validation will help translate these multi-omics insights into practical strategies for sustainable crop production in diverse environments.

## Supplementary Information

Supplementary Material 1

## Data Availability

The soybean genome data were previously processed by Kajiya-Kanegae et al. [15], and available in the DDBJ Sequence Read Archive (DRA) under the BioProject accession number PRJDB7281. The metabolome raw data were previously processed by Dang et al. [7], available on the RIKEN DropMet website (http://prime.psc.riken.jp/menta.cgi/prime/drop_index; ID: DM0071). The data are also provided as supplementary materials. The microbiome raw read sequences are available in the DDBJ DRA under the BioProject accession number PRJDB37881 and were originally generated and published in this study. The data are also provided as supplementary materials. The plant phenotype data were originally processed and analyzed in this study and The data are provided as supplementary materials. All codes and data are available at GitHub (https://github.com/Yoska393/ShapPlantMicro).

## Appendix A: Multi-omics data collection

### Field experiment

The accessions and experimental fields were the same as those utilized by Toda et al. [38], Sakurai et al. [31], Dang et al. [7]. A diverse collection of 198 soybean accessions, registered with the NARO Gen-bank (https://www.gene.affrc.go.jp/), was employed. This group primarily comprises global soybean minicore collections [15]. The field trial took place in 2019 at the Arid Land Research Center of Tottori University on sandy soil. Sowing was carried out at the beginning of July, and plants were thinned two weeks later. Prior to sowing, fertilizers (13, 6.0, 20, 11, and 7.0 g m$^{-2}$ of N, P, K, Mg, and Ca, respectively) were applied to the field. White mulch sheets (Dupont, Wilmington, DE, USA) were placed to minimize rainwater infiltration and to regulate soil conditions under artificial irrigation. Irrigation tubes were installed beneath the sheets to water the fields. Two watering regimes, non-watered and well-watered, were established to assess the effects of drought and control conditions. The watering treatment began after thinning and continued two weeks post-sowing, with artificial irrigation applied at a flow rate of 1.1 L h$^{-1}$ m$^{-1}$ for 5 h daily (7:00–9:00, 12:00–14:00, and 16:00–17:00). Phenotypic sampling (from the 2nd and 3rd individuals) and root sampling (from the 3rd individual) were performed at the beginning of September. These data were obtained from the same plot to facilitate the data integration for the model. The sampled root was stored at –80$^{\circ }$C until metabolome and microbiome analyses.

### Phenotypic traits

The biomass-related phenotypic traits measured were dry weight of leaves (LeafDryWeight), dry weight of shoots (ShootDryWeight), fresh weight of shoots (ShootFreshWeight), fresh weight of leaves (LeafFreshWeight), growth stage (GrowthStage), number of nodes (NumberOfNodes), number of branches (NumberOfBranches), plant height (PlantHeight), and main stem length (MainStemLength).

### Metabolome analysis

Metabolome analysis of root samples was conducted as follows. Samples were freeze-dried, powdered using a mixer (Shake Master NEO BMS-M10N21, Bio Medical Science, Tokyo, Japan), and 4 mg of the powder was extracted with 1 mL of solvent (80% methanol containing 0.1% formic acid, 8.4 nM lidocaine, and 210 nM 10-camphorsulfonic acid as internal standards). The mixture was sonicated for 10 min and centrifuged at 10,000 rpm for 5 min, and the supernatant was collected as the extract. To 25 $\mu$L of the extract, 75 $\mu$L of extraction solvent was added. Twenty-five $\mu$L of this dilution was dried under N$_2$ gas, reconstituted in 250 $\mu$L of deionized water, and used as the test solution. Final concentrations of the internal standards in the test solutions were 0.84 nM for lidocaine and 21 nM for 10-camphorsulfonic acid. Following filtration through a MultiScreen 386 well-plate filter (Merck Millipore, Billerica, MA, USA), metabolome analysis was performed using a Shimadzu LCMS-8050 system coupled to a Nexera X2 UPLC system (Shimadzu, Kyoto, Japan). Chromatographic separation employed a Waters HSS T3 column (1.0 $\times$ 50 mm, 1.8 $\mu$m) at 30 $^{\circ }$C. The mobile phases were 0.1% (v/v) formic acid in water (A) and 0.1% (v/v) formic acid in acetonitrile (B), with a gradient of 0.1–9% (B) at 0.25–0.4 min, 9–17% (B) at 0.4–0.8 min, 17–99.9% (B) at 0.8–1.9 min, 99.9–0.1% (B) at 1.9–2.1 min, and 0.1% (B) at 2.11–2.7 min. The flow rate was 0.24 mL/min, and the injection volume was 1 $\mu$L.

The mass spectrometer was operated in both positive and negative ESI modes. Key parameters included: nebulizing gas flow 3 mL/min, heating gas 10 L/min, drying gas 10 L/min, interface temperature 300 $^{\circ }$C, dilution line 250 $^{\circ }$C, heat block 400 $^{\circ }$C, CID gas 270 kPa, and capillary voltages of +4 kV and − 3.5 kV. Data were acquired in scheduled multiple reaction monitoring (MRM) mode for MS/MS detection, and compound-specific transitions are provided in Supplementary Table 1.

Raw data (lcd files) were converted to Abf format using the Reifycs Abf Converter (https://www.reifycs.com/AbfConverter/). Peak areas of LC–QqQ–MS data were quantified using MRMPROBS software (http://prime.psc.riken.jp/compms/mrmprobs/main.html), and peak areas were normalized based on the internal standards.

### Microbiome analysis

#### DNA extraction

Genomic DNA was isolated from root and rhizosphere soil samples following previously reported procedures. 100 mg of finely powdered sample was transferred into a 1.5 mL microtube and flash-frozen in liquid nitrogen. Subsequently, 200 $\upmu$L of lysis/binding buffer (LBB) containing 1 M LiCl (Cat. #L7026-500 ML, Sigma-Aldrich, St. Louis, USA), 100 mM Tris–HCl (Cat. #318-90225, Wako Pure Chemical Corporation, Osaka, Japan), 1% SDS (Cat. #313-90275, Wako Pure Chemical Corporation), 10 mM EDTA, pH 8.0 (Cat. #311-90075, Wako Pure Chemical Corporation), Antifoam A (Cat. #A5633-25 G, Sigma-Aldrich), 5 mM dithiothreitol (Cat. #048-29224, Wako Pure Chemical Corporation), 11.2 M 3-mercapto-1,2-propanediol (Cat. #139-16452, Wako Pure Chemical Corporation), and DNase/RNase-free water (Cat. #10977015, Thermo Fisher Scientific, Waltham, MA, USA) was added. The mixture was vortexed briefly and incubated for 5 min at room temperature (24 $^{\circ }$C).

Samples were centrifuged at 13,000 rpm for 10 min at room temperature, and 50 $\upmu$L of the supernatant was combined with an equal volume of AMPure XP beads (Cat. #A63881, Beckman Coulter, Inc.) for DNA purification and size selection. The mixture was vortexed, incubated at room temperature for 5 min, and placed on a magnetic rack for another 5 min. After discarding the supernatant, beads were washed twice with 200 $\upmu$L of 80% ethanol. DNA was eluted from the beads using 15 $\upmu$L of 10 mM Tris–HCl (pH 7.5).

#### Bacterial 16 S rRNA gene sequencing

Amplicon libraries were generated using a modified two-step PCR amplification protocol. For the initial PCR, the V4 region of the bacterial 16 S rRNA gene was amplified using primers (515f: 5’- TCG TCG GCA GCG TCA GAT GTG TAT AAG AGA CAG – [3–6-mer Ns] – GTG YCA GCM GCC GCG GTA A -3’; 806rB: 5’- GTC TCG TGG GCT CGG AGA TGT GTA TAA GAG ACA G – [3–6-mer Ns] – GGA CTA CNV GGG TWT CTA AT -3’). PCR reactions (10 $\upmu$L) contained 1 $\upmu$L of ten-fold diluted DNA template, 2$\times$ KAPA HiFi HotStart ReadyMix (Cat. #07958935001, KAPA Biosystems Inc., USA), 0.2 $\upmu$M of each primer, and 1 $\upmu$M blocking primers (mPNA and pPNA; PNA BIO, Inc., Newbury Park, CA, USA). Thermal cycling conditions were: initial denaturation at 95 $^{\circ }$C for 3 min; 35 cycles of 98 $^{\circ }$C for 20 s, 78 $^{\circ }$C for 10 s, 50 $^{\circ }$C for 30 s, and 72 $^{\circ }$C for 90 s; followed by a final extension at 72 $^{\circ }$C for 5 min (ramp rate = 1 $^{\circ }$C/s).

PCR products were purified using ExoSAP-IT Express (Cat. #75001.1.EA; Thermo Fisher Scientific). Five microliters of the PCR product were mixed with 2 $\upmu$L of ExoSAP-IT Express, incubated at 37 $^{\circ }$C for 4 min, and heat-inactivated at 80 $^{\circ }$C for 1 min.

The second PCR incorporated Illumina index sequences using primers: forward (5’- AAT GAT ACG GCG ACC ACC GAG ATC TAC AC – [8-mer index] – TCG TCG GCA GCG TC -3’) and reverse (5’- CAA GCA GAA GAC GGC ATA CGA GAT – [8-mer index] – GTC TCG TGG GCT CGG -3’). Reactions (10 $\upmu$L) included 0.8 $\upmu$L of purified first-round amplicons, 2$\times$ KAPA HiFi HotStart ReadyMix, 1 $\upmu$M of each index primer, and 1 $\upmu$M blocking primers. Cycling conditions were: 95 $^{\circ }$C for 3 min; 8 cycles of 98 $^{\circ }$C for 20 s, 78 $^{\circ }$C for 10 s, 55 $^{\circ }$C for 30 s, and 72 $^{\circ }$C for 90 s; and a final extension at 72 $^{\circ }$C for 5 min (ramp rate = 1 $^{\circ }$C/s).

Amplicons were purified using AMPure XP beads, incubated for 5 min at room temperature, and placed on a magnetic rack. After two washes with 200 $\upmu$L of 80% ethanol, DNA was eluted with 15 $\upmu$L of 10 mM Tris–HCl (pH 7.5). Library concentrations were measured using the Quant-iT PicoGreen dsDNA assay (Cat. #P7581, Invitrogen) on a microplate reader (Infinite 200 PRO M Nano+, TECAN Japan Co., Ltd., Kanagawa, Japan). Libraries were normalized and pooled in equimolar amounts, quantified by qPCR using the NEBNext Library Quant Kit for Illumina (Cat. #E7630L, New England BioLabs, USA), and diluted to 3 nM. The denatured library pool was supplemented with 20% PhiX Control v3 (Cat. #FC-110-3001, Illumina, USA) and sequenced on an Illumina MiSeq platform with the MiSeq Reagent Kit v3 (2$\times$300 bp, Illumina).

#### 16 S V4 rRNA data preprocessing

Raw paired-end reads were analyzed with QIIME2 (ver. 2020.6.0). FASTQ files were imported into QIIME2, and adapter sequences were removed with the Cutadapt plugin. For ASV inference, reads were processed with the DADA2 pipeline, which included truncation (forward reads at 240 bp, reverse reads at 180 bp), quality filtering, denoising, merging of read pairs, and chimera removal. Taxonomic assignment of amplicon sequence variants (ASVs) was performed using the SILVA database (ver. 138). Sequences assigned to Archaea, Eukaryota, mitochondria, or chloroplasts were excluded from downstream analyses.

### Multi-omics integration and scaling

For Random Forest (RF) modeling, the genome, metabolome, and microbiome features were concatenated into a single input matrix without enforcing equal feature numbers across omics layers. This integration strategy was adopted to support the primary objective of this study, which was to investigate the coordination and interaction patterns among different omics layers, rather than to compare or optimize predictive performance.

As the number of genome-wide SNP markers was substantially larger than that of the other omics layers, linkage disequilibrium (LD) pruning was applied as a standard genome analysis procedure. This reduced the original set of 425,858 SNPs to 3,078 representative markers (LD threshold = 0.001), which were broadly distributed across the genome, providing approximate genome-wide coverage and a certain prediction performance (Figs. 5,6).

Microbiome features were scaled prior to modeling, and variables with missing values or zero variance within each condition were removed by filtering. The control and drought-treated samples were preprocessed using an identical pipeline to ensure consistency. Metabolome features were similarly scaled, and all variables that passed the quality control were retained.

Differences in feature dimensionality and scale across omics layers were not explicitly corrected. Enforcing an artificial balance among omics types would require imposing arbitrary constraints and restricting the diversity of the biological signals captured. Instead, RF was used to allow for intrinsic data-driven feature selection based on the feature importance scores.

Informative features from all omics layers were retained in the downstream analyses, indicating that no single omics layer dominated the model solely because of feature dimensionality. The comparison with BLUP supported the fact that RF exhibited stable behavior across heterogeneous omics scales (Fig. 1), which is consistent with the study’s emphasis on cross-omics coordination and interaction analyses.

## Appendix B: Supplementary figures and table

See Figs. 5, 6, 7, 8, 9, 10, 11, 12, 13, 14, 15 and 16 and Tables 1, 2 and 3

**Table 1:** Effect of the threshold $\theta$ on the number of selected features and remaining edges. Here, $\theta$ denotes the threshold applied to the RF importance scores. The column “Number of Features” represents the number of features whose importance exceeds the threshold. The SHAP interaction network was then restricted to these selected features, and “Number of Edges” denotes the number of edges in the resulting SHAP subnetwork ($|Z| \ge 30$). The ratio represents the proportion of remaining edges relative to the baseline network obtained at $\theta = 0.001$ (160 edges)

$\theta$	Number of features	Number of edges	(Ratio)
0.001	2729	160	(1.00)
0.002	1341	153	(0.96)
0.003	877	146	(0.91)
0.004	677	140	(0.88)
0.005	539	126	(0.79)
0.006	454	113	(0.71)
0.007	361	103	(0.64)
0.008	277	96	(0.60)
0.009	216	85	(0.53)
0.010	159	66	(0.41)

**Table 2:** SHAP interaction edges with positive Z-scores ($Z \ge 30$). *weight* denotes the interaction difference $|\Delta |$ (Drought − Control), with two-sided *p* values and bootstrap stability

target_label	partner_label	weight	z	*p* value	Stability
Chr4_22035062	GABA	6.5e−03	559.45	9.9e−03	14
Asparagine	Daidzin	3.8e−03	326.99	9.9e−03	13
Chr13_28130797	Daidzin	3.4e−03	291.96	9.9e−03	4
Naringenin-7-O-glucoside	Mannitol	2.5e−03	215.48	9.9e−03	4
Glucoheptose	4-Hydroxybenzoate	1.9e−03	166.31	9.9e−03	7
Bacillus	Succinic acid	1.9e−03	159.76	9.9e−03	3
3-Hydroxyanthranilic acid	Daidzin	1.7e−03	148.86	9.9e−03	13
2,3-Diaminopropionic acid	Aspartic acid	1.7e−03	146.13	9.9e−03	2
Bacillus	Carbamoyl-DL-aspartic acid	1.5e−03	130.88	2.0e−02	9
Sphingobacterium	Quinic acid	1.3e−03	114.72	9.9e−03	2
Ramlibacter	CarnitineHCl	1.3e−03	114.12	9.9e−03	2
Anthranilic acid	Daidzin	1.3e−03	113.07	9.9e−03	10
Ornithine	Daidzin	1.3e−03	112.99	2.0e−02	15
Glucoheptose	(+-)-Jasmonic acid	1.3e−03	112.00	9.9e−03	12
Streptomyces	Paenibacillus	1.3e−03	110.09	9.9e−03	2
Pseudoflavitalea	Malic acid	1.3e−03	108.44	9.9e−03	3
Duganella	Chr18_41720702	1.2e−03	104.01	9.9e−03	2
Genistin	Genistein	1.2e−03	102.08	9.9e−03	3
6”-O-Acetylgenistin	Chr3_39862107	1.1e−03	94.72	9.9e−03	2
Chr4_22035062	Tahibacter	1.1e−03	93.01	9.9e−03	3
Chr3_7600737	Chr3_27222972	1.1e−03	92.17	9.9e−03	2
Paenibacillus	Daidzin	1.1e−03	92.10	9.9e−03	6
Chr1_32578572	Asparagine	1.1e−03	91.76	9.9e−03	2
Uridine	6”-O-Acetylgenistin	1.1e−03	90.91	9.9e−03	2
2,3-Diaminopropionic acid	Glutamic acid	1.1e−03	90.53	9.9e−03	4
Bacillus	Glucoheptose	1.0e−03	86.63	9.9e−03	9
Chr1_32578572	Daidzin	1.0e−03	86.12	9.9e−03	8
Anserine	Daidzin	9.8e−04	83.84	9.9e−03	10
Daidzin	6”-O-Malonylglycitin	9.6e−04	82.24	9.9e−03	3
Streptomyces	Zeatin	9.2e−04	79.34	9.9e−03	2
2,3-Diaminopropionic acid	Daidzin	9.2e−04	78.85	9.9e−03	26
uncultured	Tryptophane	9.0e−04	77.19	9.9e−03	5
GABA	Mannitol	8.9e−04	76.78	9.9e−03	3
Paenibacillus	GABA	8.8e−04	75.35	9.9e−03	3
Chr4_22035062	Carbamoyl-DL-aspartic acid	8.8e−04	75.27	9.9e−03	3
Asparagine	Indole-3-carboxyaldehyde	8.4e−04	71.89	9.9e−03	8
Ornithine	Pelargonin chloride	8.2e−04	70.49	9.9e−03	4
Asparagine	Suberic acid	7.7e−04	65.74	9.9e−03	9
Chr1_42816170	Pelargonin chloride	7.4e−04	63.95	9.9e−03	4
Chr3_27222972	Ramlibacter	7.4e−04	63.13	9.9e−03	3
Anthranilic acid	Orobol	7.3e−04	62.65	9.9e−03	3
GABA	Apiin	7.1e−04	60.63	9.9e−03	13
Glucoheptose	5-Hydroxylysine hydrochloride	7.0e−04	60.25	9.9e−03	11
Threonic acid hemicalcium salt	Salicylic Acid	7.0e−04	59.70	9.9e−03	13
Niastella	Glucoheptose	6.9e−04	59.53	9.9e−03	3
Bacillus	4-Hydroxybenzoate	6.9e−04	59.34	9.9e−03	2
Chr1_12369026	Chr3_27222972	6.4e−04	54.89	9.9e−03	15
Bacillus	3,4-Dihydroxy-L-phenylalanine	6.4e−04	54.76	9.9e−03	2
Flavanomarein	Daidzin	6.3e−04	54.10	9.9e−03	4
Rhodanobacter	Daidzin	6.2e−04	53.57	9.9e−03	6
Chr4_22035062	Arginine	6.2e−04	52.98	9.9e−03	2
Ornithine	Sodium pantothenate	5.6e−04	48.08	9.9e−03	5
Streptomyces	Aminobutyric acid	5.6e−04	47.96	9.9e−03	4
-Phenylalanine	Chr3_39862107	5.5e−04	46.81	9.9e−03	4
Chr1_32578572	2’-Hydroxydaidzein	5.4e−04	46.75	9.9e−03	3
Ramlibacter	3-Hydroxyanthranilic acid	5.4e−04	46.48	9.9e−03	4
Anthranilic acid	Anserine	5.4e−04	46.44	9.9e−03	6
-Phenylalanine	Uridine	5.3e−04	45.93	9.9e−03	7
Chr11_1888757	Daidzin	5.2e−04	44.79	9.9e−03	7
Nicotinic acid mono nucleotide	Fructose-6-phosphate	5.2e−04	44.68	9.9e−03	9
Amantadine hydrochloride	Chr12_8480223	5.1e−04	43.96	9.9e−03	5
Chr6_6448277	Fructose-6-phosphate	5.1e−04	43.55	9.9e−03	3
1,1-Dimethylbiguanide hydrochloride	6”-O-Acetylgenistin	5.1e−04	43.54	9.9e−03	5
Novosphingobium	Glucoheptose	4.9e−04	42.07	9.9e−03	2
Threonic acid hemicalcium salt	6”-O-Malonylgenistin	4.9e−04	42.07	9.9e−03	5
Succinic acid	4-Hydroxybenzoate	4.8e−04	41.64	9.9e−03	3
Threonic acid hemicalcium salt	Daidzin	4.8e−04	41.35	9.9e−03	19
Chr4_22035062	1-Methylhistamine dihydrochloride	4.7e−04	40.23	9.9e−03	4
Actinomadura	-Phenylalanine	4.6e−04	39.73	9.9e−03	9
Ala-Ala	Arginine	4.4e−04	37.70	9.9e−03	3
3-Methyl-L-histidine	Daidzin	4.4e−04	37.69	9.9e−03	14
Myricetin-3-Galactoside	Indole-3-carboxyaldehyde	4.3e−04	36.73	9.9e−03	3
Duganella	Amantadine hydrochloride	4.3e−04	36.59	9.9e−03	7
uncultured	Puia	4.2e−04	36.06	9.9e−03	3
Aminobutyric acid	Malic acid	4.1e−04	34.90	9.9e−03	3
uncultured	Asparagine	4.0e−04	34.77	9.9e−03	3
Chr1_5452592	Chr4_22035062	4.0e−04	34.20	9.9e−03	21
Chr1_5083560	2’-Hydroxydaidzein	3.8e−04	32.83	9.9e−03	23
Asparagine	3-Methyl-L-histidine	3.8e−04	32.55	9.9e−03	12
Aquisphaera	GABA	3.7e−04	32.02	9.9e−03	2
Asparagine	Quinic acid	3.7e−04	32.00	2.0e−02	2
6”-O-Acetylgenistin	Chr7_35929840	3.7e−04	31.97	9.9e−03	2
Chr4_22035062	Ala-Ala	3.7e−04	31.89	9.9e−03	5
Chr3_27222972	3-Hydroxyanthranilic acid	3.7e−04	31.46	9.9e−03	12
Streptomyces	2,3-Diaminopropionic acid	3.6e−04	31.33	9.9e−03	2
Myricetin-3-Galactoside	Aminobutyric acid	3.6e−04	30.97	9.9e−03	4
Chr10_44957783	Cystathionine	3.6e−04	30.94	9.9e−03	2
GABA	Indole-3-carboxyaldehyde	3.6e−04	30.72	2.0e−02	8
uncultured	Bacillus	3.6e−04	30.49	9.9e−03	5
Chr3_27222972	Labrys	3.5e−04	30.05	9.9e−03	7

**Table 3:** SHAP interaction edges with negative Z-scores ($Z \le -30$). *weight* denotes the interaction difference $|\Delta |$ (Drought − Control), with two-sided *p* values and bootstrap stability

target_label	partner_label	weight	z	*p* value	Stability
Asparagine	Synephrine	3.8e−03	− 322.72	9.9e−03	12
Succinic acid	Glucoheptose	3.4e−03	− 290.88	2.0e−02	4
Chr13_28130797	Suberic acid	3.2e−03	− 276.77	9.9e−03	2
Chr2_15739835	Apiin	2.8e−03	− 243.16	9.9e−03	12
Cystathionine	Serine	2.8e−03	− 238.25	9.9e−03	7
Chr4_22035062	Bacillus	1.8e−03	− 155.80	9.9e−03	12
Chr4_22035062	Succinic acid	1.4e−03	− 123.08	9.9e−03	3
Chr3_27222972	Ornithine	1.4e−03	− 121.02	9.9e−03	11
Quinic acid	Salicylic Acid	1.4e−03	− 120.89	9.9e−03	6
Chr4_22035062	Pimelic acid	1.3e−03	− 112.57	9.9e−03	9
Cystathionine	Methylaspartic acid	1.3e−03	− 107.60	9.9e−03	3
Niastella	Asparagine	1.2e−03	− 102.17	9.9e−03	2
2,3-Diaminopropionic acid	Melibiose hydrate	1.2e−03	− 100.95	9.9e−03	2
Chr4_22035062	Chr12_8480223	1.2e−03	− 99.56	9.9e−03	6
2,3-Diaminopropionic acid	Anserine	1.1e−03	− 93.72	9.9e−03	2
Chr1_4873925	Chr10_44957783	1.1e−03	− 92.84	9.9e−03	6
Asparagine	2’-Hydroxydaidzein	1.0e−03	− 85.78	9.9e−03	11
Gemmatimonas	GABA	9.8e−04	− 84.33	9.9e−03	2
Pelargonin chloride	Daidzin	9.4e−04	− 80.95	9.9e−03	6
1-Aminocyclopropane-1-carboxylic acid	GABA	9.2e−04	− 79.34	9.9e−03	2
GABA	Pimelic acid	9.1e−04	− 77.77	9.9e−03	14
uncultured	Glucoheptose	8.9e−04	− 76.64	9.9e−03	3
Chr4_22035062	Glucoheptose	8.8e−04	− 75.83	9.9e−03	11
Chr3_27222972	Conexibacter	8.6e−04	− 74.12	9.9e−03	4
Indole-3-carboxyaldehyde	Daidzin	8.6e−04	− 73.86	9.9e−03	17
Chr1_4873925	Glucoheptose	8.6e−04	− 73.55	9.9e−03	38
Chr4_7828113	Chr4_22035062	8.5e−04	− 73.08	9.9e−03	4
Chr4_22035062	Terrabacter	8.4e−04	− 71.76	9.9e−03	2
Chr10_45418069	Fructose-6-phosphate	8.3e−04	− 71.41	9.9e−03	6
Salicylic acid	Daidzin	8.2e−04	− 70.70	2.0e−02	4
Suberic acid	3-Methyl-L-histidine	7.9e−04	− 68.07	9.9e−03	6
Ornithine	Apiin	7.8e−04	− 67.30	9.9e−03	5
Chr3_27222972	Pelargonin chloride	7.7e−04	− 66.38	9.9e−03	6
Cystathionine	Quinic acid	7.5e−04	− 64.61	9.9e−03	8
Duganella	Terrabacter	7.5e−04	− 64.56	9.9e−03	2
Chr1_33799527	Chr18_41720702	7.4e−04	− 63.74	9.9e−03	2
GABA	Naringenin-7-O-glucoside	7.4e−04	− 63.35	9.9e−03	11
Chr4_22035062	Amantadine hydrochloride	7.1e−04	− 60.96	9.9e−03	7
Paenibacillus	Asparagine	7.0e−04	− 60.52	9.9e−03	3
Chr1_4873925	Chr17_9067041	7.0e−04	− 60.24	9.9e−03	3
Chr1_10557956	Guanosine	6.9e−04	− 58.88	9.9e−03	7
1,1-Dimethylbiguanide hydrochloride	Uridine	6.5e−04	− 55.94	9.9e−03	9
Streptomyces	6”-O-Acetyldaidzin	6.3e−04	− 53.75	9.9e−03	2
uncultured	Fructose-6-phosphate	6.1e−04	− 52.51	9.9e−03	2
Duganella	Chr12_8480223	6.1e−04	− 52.14	9.9e−03	2
Jatrophihabitans	Daidzin	5.9e−04	− 50.75	9.9e−03	6
Tryptophane	6”-O-Acetylgenistin	5.9e−04	− 50.28	9.9e−03	4
uncultured	Daidzin	5.7e−04	− 48.83	9.9e−03	5
Argininosuccinic acid disodium salt	Naringenin-7-O-glucoside	5.6e−04	− 48.43	9.9e−03	3
Daidzin	Orobol	5.2e−04	− 44.65	9.9e−03	2
GABA	2-Aminoadipic Acid	5.1e−04	− 43.87	9.9e−03	4
Asparagine	Luteolin	5.1e−04	− 43.76	9.9e−03	2
Peonidin-3,5-O-di-beta-glucopyranoside	Daidzin	5.1e−04	− 43.54	9.9e−03	3
Quinic acid	Daidzin	4.9e−04	− 42.25	9.9e−03	18
2,3-Diaminopropionic acid	3-Hydroxyanthranilic acid	4.8e−04	− 41.09	9.9e−03	15
Mycobacterium	Bacillus	4.6e−04	− 39.78	9.9e−03	3
Quinic acid	3-Methyl-L-histidine	4.6e−04	− 39.63	9.9e−03	4
Jatrophihabitans	Asparagine	4.6e−04	− 39.53	9.9e−03	3
Pseudoflavitalea	Threonic acid hemicalcium salt	4.5e−04	− 38.84	9.9e−03	2
Abscisic acid	Fructose-6-phosphate	4.5e−04	− 38.66	9.9e−03	4
3-Hydroxyanthranilic acid	Flavanomarein	4.5e−04	− 38.56	9.9e−03	6
Fructose-6-phosphate	Genistin	4.5e−04	− 38.43	9.9e−03	3
Devosia	GABA	4.4e−04	− 37.80	9.9e−03	2
Malic acid	Chr12_8480223	4.3e−04	− 36.52	9.9e−03	6
Rhodanobacter	Ornithine	4.1e−04	− 35.06	9.9e−03	7
2,3-Diaminopropionic acid	Orobol	4.0e−04	− 34.61	9.9e−03	3
Suberic acid	Daidzin	4.0e−04	− 34.45	9.9e−03	13
Ramlibacter	Daidzin	3.9e−04	− 33.92	9.9e−03	10
Ramlibacter	2,3-Diaminopropionic acid	3.8e−04	− 32.54	9.9e−03	4
Succinic acid	Malic acid	3.8e−04	− 32.43	2.0e−02	3
Indole-3-carboxyaldehyde	2’-Hydroxydaidzein	3.6e−04	− 30.79	9.9e−03	15

## Appendix C: Implementation

### Analysis

Statistical analyses and data visualizations were performed using a combination of R (version 4.4.1) and Python (version 3.10.9, Clang 14.0.6). Figures created in R primarily used the ggplot2 package (version 3.5.1), while Python-based analyses–including SHAP value interpretation and XGBoost modeling–were conducted via the reticulate interface in RStudio. All the codes are available on GitHub: (https://github.com/Yoska393/ShapPlantMicro)

### Hyperparameter tuning

We implemented two Random Forest (RF) models for phenotypic prediction using omics data. RF is a robust machine learning method that reduces overfitting through bootstrapping. It includes several hyperparameters, such as tree depth and the number of trees, which influence performance.

The first model used the ranger implementation [44] to predict biomass-related phenotypes based on the full omics dataset (8,757 predictors). Each response was modeled separately, and the mean performance was reported.

- mtry $\in \left\{ \left\lfloor \frac{\text {ncol}(X)}{6} \right\rfloor , \left\lfloor \frac{\text {ncol}(X)}{3} \right\rfloor , \left\lfloor \frac{\text {ncol}(X)}{1.5} \right\rfloor \right\}$: the number of variables randomly sampled as candidates at each split. Smaller values introduce more randomness and can help prevent overfitting.
- min.node.size $\in \{3, 5, 7\}$: the minimum number of observations in a terminal (leaf) node. Smaller values allow the model to grow deeper trees, potentially capturing more detail but increasing the risk of overfitting.
- num.trees $\in \{100, 500, 1000\}$: the total number of trees in the forest. More trees generally improve stability and performance, but increase computation time.

The second model used XGBoost [6] for predicting a representative dry weight phenotype using a reduced feature set (2729 features). The variable inputs were selected based on feature importance scores from the first model. The XGBoost hyperparameter grid included:

- learning_rate $\in \{0.1, 0.3, 0.5\}$: the step size shrinkage used in each boosting step. Lower values make the model more robust but require more boosting rounds.
- n_estimators $\in \{50, 100, 500\}$: the number of boosting rounds (trees). More rounds can improve performance, but may lead to overfitting if not regularized.
- max_depth $\in \{3, 6, 9\}$: the maximum depth of each tree. Shallower trees are more regularized and less prone to overfitting, while deeper trees can model complex patterns.

Both models were evaluated using 10-fold cross-validation under control and drought conditions, with mean squared error (MSE) as the evaluation metric. To ensure a fair comparison, we aimed to select identical hyperparameters for both conditions whenever possible.

The grid search results, summarized in Fig. 7, show minimal differences in performance across all hyperparameters. RF has proven robust under all tested configurations, likely because of its bootstrapping strategy. We object to selecting the same hyperparameter set, which is stable under both control and drought conditions. Considering overall performance, overfitting risks, and computational efficiency, we selected the following default configurations:

- *Ranger* mtry = $\left\lfloor \frac{\text {ncol}(X)}{3} \right\rfloor$, min.node.size = 5, num.trees = 500
- *XGBoost* learning_rate = 0.3, n_estimators = 100, max_depth = 6

### Stability-based filtering of differential interactions

Under the present modeling and feature-selection setting, SHAP interaction matrices are extremely sparse. In a single bootstrap replicate, the observed edge density was approximately $0.015\%$ (5,563 edges out of 3,722,356 possible feature pairs).

As a rough baseline, the appearance of a specific interaction edge in a given bootstrap replicate can be treated as a rare event with probability $p \approx 1.5 \times 10^{-4}$. Under this assumption, the number of times an edge is observed across $B_{\textrm{boot}} = 100$ bootstrap replicates can be approximated by a binomial distribution:

$$ X \sim \textrm{Binomial}(100, p). $$

Although it may appear counterintuitive, this null model implies that repeated detection of the same edge, even in a small number of bootstrap replicates, is already highly unlikely to occur by chance alone. Specifically, the probabilities that a given edge appears in at least two, three, or four Bootstrap replicates are given by

$$ \Pr (X \ge 2) = 1 - \sum _{k=0}^{1} \left( {\begin{array}{c}100\\ k\end{array}}\right) p^k (1-p)^{100-k} \approx 1.1 \times 10^{-4}, $$

$$ \Pr (X \ge 3) = 1 - \sum _{k=0}^{2} \left( {\begin{array}{c}100\\ k\end{array}}\right) p^k (1-p)^{100-k} \approx 5.4 \times 10^{-7}, $$

and

$$ \Pr (X \ge 4) = 1 - \sum _{k=0}^{3} \left( {\begin{array}{c}100\\ k\end{array}}\right) p^k (1-p)^{100-k} \approx 2.0 \times 10^{-9}. $$

Thus, observing the same interaction in only 2–4 out of 100 bootstrap replicates already provides non-trivial statistical evidence against a purely random, sparsity-driven null model.

Based on this reasoning, we adopted a conservative stability-based filtering criterion requiring an edge to be observed in at least two bootstrap replicates ($n^{\textrm{obs}}_{ij} \ge 2$). This threshold effectively removes isolated, non-reproducible edges that are likely to arise from resampling noise, while retaining interactions that are consistently recovered across bootstrap samples.
